# BID-seq for transcriptome-wide quantitative sequencing of mRNA pseudouridine at base resolution

**DOI:** 10.1038/s41596-023-00917-5

**Published:** 2023-11-15

**Authors:** Li-Sheng Zhang, Chang Ye, Cheng-Wei Ju, Boyang Gao, Xinran Feng, Hui-Lung Sun, Jiangbo Wei, Fan Yang, Qing Dai, Chuan He

**Affiliations:** 1 Department of Chemistry, The University of Chicago, Chicago, IL, USA.; 2 Howard Hughes Medical Institute, The University of Chicago, Chicago, IL, USA.; 3 Division of Life Science, The Hong Kong University of Science and Technology, Hong Kong SAR.; 4 Department of Chemistry, The Hong Kong University of Science and Technology, Hong Kong SAR.; 5 Pritzker School of Molecular Engineering, The University of Chicago, Chicago, IL, USA.; 6 Department of Human Genetics, The University of Chicago, Chicago, IL, USA.; 7 Department of Biochemistry and Molecular Biology, The University of Chicago, Chicago, IL, USA.; 8 Institute for Biophysical Dynamics, The University of Chicago, Chicago, IL, USA.

## Abstract

Pseudouridine (Ψ) is an abundant RNA modification that is present in and impacts the functions of diverse non-coding RNA species, including rRNA, tRNA, snRNA, etc. Pseudouridine also exists in mammalian mRNA and indicates functional roles; however, functional investigations of pseudouridine in mammalian mRNA have been hampered by the lack of a quantitative method that detects Ψ at base precision. We have recently developed Bisulfite-Induced Deletion sequencing (BID-seq), which provides the community with a quantitative method to map RNA Ψ distribution transcriptome-wide at single-base resolution. Here, we describe an optimized BID-seq protocol to generate highly reproducible results, displaying nearly zero background deletions at unmodified uridines after bisulfite treatment and uncovering 8,407 Ψ sites starting from as little as 10 ng mouse embryonic stem cell (mESC) polyA^+^ RNA, with the steps that are fast in both library preparation and data analysis. This optimized BID-seq workflow takes 5 days to complete and includes four main sections: RNA preparation, library construction, next-generation sequencing (NGS), and data analysis. The library construction can be completed by researchers who have basic knowledge and skills in molecular biology and genetics. In addition to the experimental protocol, we provide BID-pipe (https://github.com/y9c/pseudoU-BIDseq), a user-friendly data analysis pipeline for BID-seq, requiring only basic bioinformatic and computational skills to uncover Ψ signatures from NGS data.

## Introduction

Posttranscriptional RNA modifications occur in almost all RNA species across most life forms. In mammals, *N*^6^-methyladenosine (m^6^A) broadly regulates mRNA fate, including stability, nuclear export, pre-mRNA processing and translation^1–3^. Besides internal m^6^A methylation, pseudouridine (Ψ) ^4–9^, 2’-*O*-methylation (N_m_) ^10^, 5-methylcytidine (m^5^C) ^11–13^, *N*^1^-methyladenosine (m^1^A) ^14–17^, internal *N*^7^-methylguanosine (m^7^G) ^18,19^, and *N*^4^-acetylcytidine (ac4C) ^20,21^ have been recently reported as mammalian mRNA modifications. Among these mRNA modifications, m^6^A and ψ are the two most abundant, displaying ~0.4–0.6% m^6^A/A ^22,23^ and ~0.2% ψ /U ^7,8^ levels in mRNA based on mass spectrometry measurement. Other mRNA modifications, such as N_m_, m^5^C, m^1^A, and internal m^7^G tend to be present at lower levels.

Investigations of m^6^A biological functions so far have mostly employed m^6^A-MeRIP-seq ^22,23^, which has facilitated recent extensive research on RNA m^6^A ^1–3^. By contrast, functional investigations of mRNA ψ have proceeded at a much slower pace due to the lack of a quantitative method that detects ψ transcriptome-wide. The detection of mRNA ψ previously relied on its reaction with *N*-cyclohexyl-*N*’-(2-morpholinoethyl)carbodiimide methyl-*p*-toluenesulfonate (CMC) to generate CMC-modified ψ^4–6^, which leads to truncation signatures during reverse transcription (RT). CMC-based approaches only detected a modest number of ψ sites in human mRNA, with a low overlap among independent datasets. An azide-modified CMC has been used to enrich ψ-containing mRNA fragments for sequencing and can capture more ψ sites ^7^; however, this approach does not allow the collection of stoichiometry information.

Taking advantage of the recently reported bisulfite chemistry on ψ^24–26^, we developed Bisulfite-Induced Deletion sequencing (BID-seq) ^9^ which can efficiently and quantitatively map ψ transcriptome-wide. We demonstrated that BID-seq stoichiometrically converts ψ to the ψ-bisulfite (ψ-BS) adduct without detectable C-to-U conversion, and SuperScript IV reverse transcriptase (SuperScript IV RT) reads out high deletion rates at the BS-modified ψ sites in almost all sequence contexts of NNΨNN during RT ^9^. With spike-in probes containing varied ψ levels to calibrate sequence-context-dependent deletion rate, we were further able to calculate the stoichiometry at each ψ-modified site^9^.

We first applied BID-seq to cellular total RNA from HeLa cells, and validated almost all ψ modified sites in 18S, 28S, and 5.8S rRNA. We next applied BID-seq to polyA^+^ RNA samples and detected thousands of ψ sites (with ψ modification stoichiometry above 10%) in 3 human cell lines and 12 mouse tissues ^9^. BID-seq monitors ψ stoichiometry changes and assigns different ‘writer’ proteins to individual ψ sites, revealing TRUB1 as the main ψ ‘writer’ protein that regulates mRNA stability ^9^. BID-seq also confirmed the presence of ψ within mRNA stop codons, which promote stop codon read-through *in vivo* ^9^. BID-seq paves the way for future investigations of biological functions of ψ in mRNA and other RNA species.

Here we describe a further optimized BID-seq protocol by applying it to polyA^+^ RNA and introduce BID-pipe, a user-friendly pipeline for BID-seq data analysis to demonstrate the outstanding performance of BID-seq in ψ detection and quantification.

### Overview of the BID-seq procedure

**Fig. 1 |:**
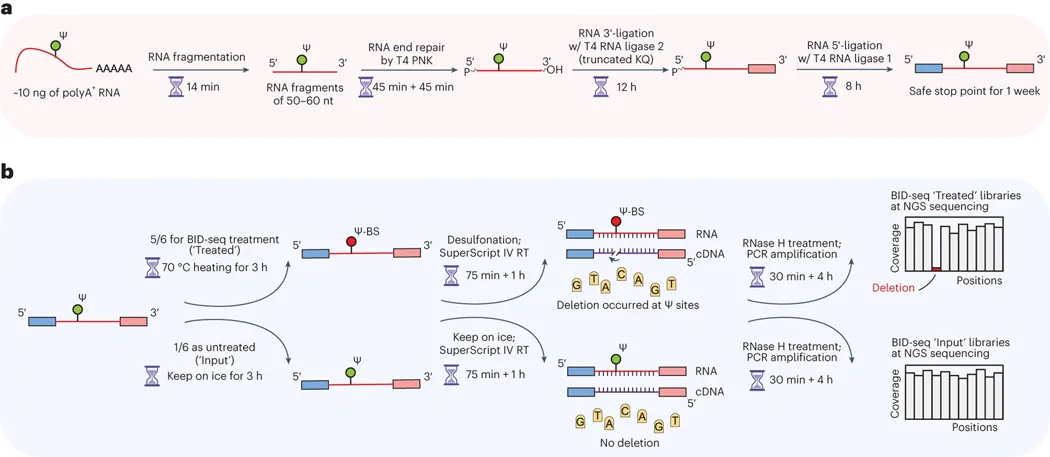
The library preparation steps for BID-seq. **a**, Stage 1 is to prepare 5’- and 3’-ligated RNA fragments from purified polyA^+^ RNA, with ψ sites marked as green circles. **b**, Stage 2 is to induce deletion signatures at internal ψ sites through BID-seq bisulfite treatment and SuperScript IV RT, with ψ -BS adduct marked as red circles.

The entire BID-seq procedure consists of two stages (Fig. 1). The first stage is the preparation of ligated RNA fragments (Fig. 1a), which involves the preparation of ~10 ng polyA^+^ RNA and RNA fragmentation (30 min), RNA 3’- and 5’-end repair by the T4 PNK enzyme (45 min + 45 min, respectively); RNA 3’-ligation (12 hours) and RNA 5’-ligation (8 hours). After this stage, the protocol reaches a stop point where the ligated RNA can be stored at −80 °C for up to a week.

The second stage induces deletion signatures at ψ-modified sites (Fig. 1b. This involves the BID-seq bisulfite treatment (3 hours), RNA desulphonation (75 min), SuperScript IV RT reaction (1 hour), RNase H treatment (30 min), PCR amplification (1 hour), Next-generation sequencing (NGS) and BID-seq data analysis using BID-pipe. Note that the on-column RNA desulphonation has been extended from 60 min ^9^ to 75 min to ensure robust and complete desulphonation at unmodified uridines, which is important for removing background noise.

### Development of the protocol

#### BID-seq bisulfite chemistry

When mapping m^5^C in RNA using RBS-seq (a modified version of RNA bisulfite sequencing ^24^), Khoddami *et al*. observed that bisulfite treatment causes modest base deletions at ψ sites in RNAs ^24^, and ψ-BS adducts were characterized as the key intermediates to trigger deletion signatures during RT ^25,26^. RBS-seq provided a new principle for ψ detection other than CMC-based methods ^4–7^. However, RBS-seq provides moderate efficiency in generating ψ-BS adduct, and thus only detects a modest number of ψ sites with deletion rates above 5% (15, 20, and 78 ψ sites in human 18S rRNA, 28S rRNA, and mRNA, respectively) ^24^. In addition, the bisulfite treatment in RBS-seq inevitably converted all the cytidines into uridines; resulting in reduced read complexity and mapping issues.

In our previously published work ^9^, we noticed that, under acidic conditions (pH ~5.1), the protonation of N3 in cytosine is critical to bisulfite-mediated deamination, while a neutral pH is more suitable for the bisulfite reaction to occur at uracil. We reasoned that neutral pH should inhibit the undesired C to U conversion but promote the bisulfite reaction at ψ to yield ψ-BS adduct. Indeed, the bisulfite treatment of a model RNA probe at neutral pH offered an almost quantitative conversion of ψ to ψ-BS adduct without any detectable C to U conversion ^9^. We then used a 30-mer ψ-containing RNA probe containing NN ψ NN motifs to screen reverse transcriptase to generate deletions at the ψ site. We found that SuperScript IV RT could generate high deletion readout at the fully modified ψ site and named this new method Bisulfite-Induced Deletion sequencing (BID-seq). Note that deletion rates were very low at unmodified bases (A, C, G, U), indicating a very low background. The level of C-to-U conversion at C bases was also very low in both ‘input’ and ‘BID-seq’ libraries, avoiding the mapping issues in RBS-seq. As a result, BID-seq validated almost all ψ modified sites in 18S, 28S, and 5.8S rRNA, and detected several thousand ψ sites in human cell lines and mouse tissues ^9^.

#### Detection of deletion signatures induced by ψ modification

Deletions are sensitive signatures for detecting RNA modifications compared with misincorporations and truncations. Firstly, the basal deletion ratio in gene regions is 10 times lower than the substitution mutation ratio. Secondly, in the Illumina high-throughput sequencing platform, the error rate of base deletion is 1 to 2 orders of magnitude lower than the probability of base substitution. These result in extremely low background noise for BID-seq. Using deletions to detect ψ is more specific than using truncation signatures, which are affected by RNA secondary structure, GC content, alternative splicing, or RNA fragmentation, which makes BID-seq much more accurate for detecting ψ compared to CMC-based methods ^4–7^.

**Fig. 2 |:**
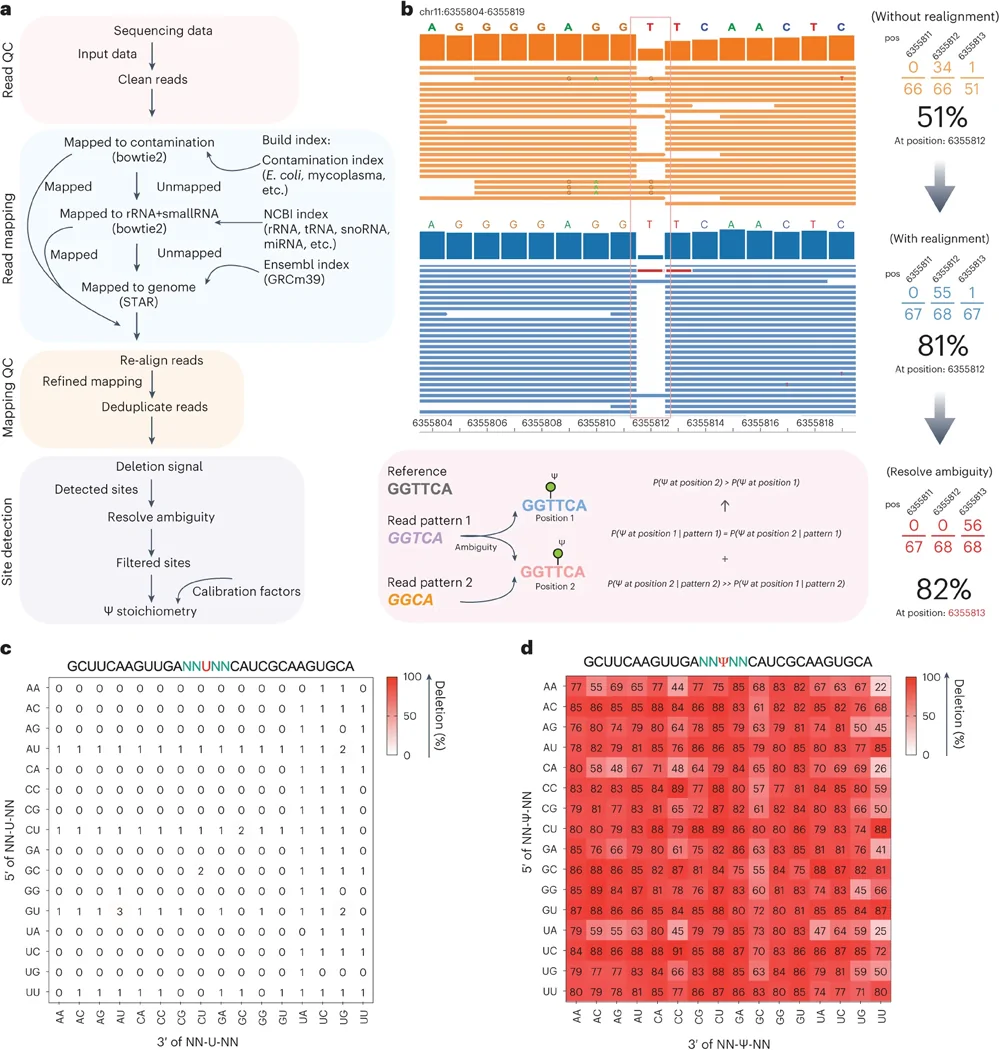
The enhanced data analysis pipeline for BID-seq with realignment technology, termed BID-pipe. **a**, The flow chart of BID-pipe pipeline in analyzing BID-seq NGS data. **b**, An example of a highly modified mRNA ψ site to show how realignment enhances deletion detection and ambiguity elimination. Left: The upper orange graph is the IGV plot without realignment, while the lower blue graph shows the reads coverage after realignment. The pink box highlighted the ψ site. Right: The numerator and denominator indicate the deletion count and total reads coverage at each position respectively, with the percentage showing the calculated deletion ratio at each position. **c**, Heatmap plot for deletion ratios on 256 motifs (NNUNN) after bisulfite treatment in BID-seq, which contain one 0% modified Ψ within each motif. **d**, Heatmap plot for deletion ratios on 256 motifs (NNΨNN) after bisulfite treatment in BID-seq, which contain one 100% modified ψ within each motif.

In this protocol, we introduce the BID-pipe protocol to sensitively uncover deletion signals across the whole transcriptome (Fig. 2a). We use realignment strategies to enhance deletion detection; briefly, we adopted two rounds of alignment and fine-tuned the parameters in the alignment software to make it more sensitive and robust for reads that contain gap signatures. Reads are first aligned to rRNA, tRNA, snoRNA, snRNA, and repeats RNA, which are of multiple copies and carry highly modified ψ, and then aligned to the genome. This can effectively reduce errors in alignment and genome annotation, which are prone to occur in repetitive sequences. It also avoids overestimating the modification level of mRNA ψ sites due to homologous sequences.

Alignment software usually uses a seed alignment algorithm to increase alignment speed; however, this also affects pairwise alignment accuracy, especially for bases near deletion signatures. To solve this, we integrated the Smith-Waterman local alignment algorithm into the pipeline for realignment. Reads that contain any mismatch, deletion, insertion, soft-clip, or splicing are further processed by the realignment tool in the BID-pipe package. By setting the penalty of gap open and gap extension as −3 and −2, respectively, deletion signatures can have a higher priority in the alignment. Using a highly modified mRNA Ψ site within GGUUCA motif as an example, the deletion ratio calculated at the gap site can be notably improved by the realignment analysis, increasing from ~51% to ~82% (Fig. 2b). The realignment in BID-pipe analysis uses the “double gaps” signature to resolve the ambiguity of the ψ position within motif contexts containing two successive uridines, and determine which U within GGUUCA is ψ-modified (Fig. 2b).

### Applications of BID-seq

This BID-seq protocol enables the mapping of transcriptome-wide ψ profiles starting from ~20,000 cells, or ~10 ng polyA^+^ RNA (from ~500 ng cellular total RNA). Unlike traditional MeRIP-seq methods ^20,21^, BID-seq does not require antibody-based pulldown enrichment or an RNA input of more than several hundred nanograms (ng). The bisulfite chemistry of BID-seq, which employs inexpensive chemicals, can be reproduced robustly by researchers with basic knowledge and skills in molecular biology and genomics. Although BID-seq is a bisulfite-based method, it can start with very low RNA input, since its bisulfite chemistry has been optimized to be mild and efficient enough to avoid dramatic RNA degradation. Besides abundant RNA such as rRNA, BID-seq is also powerful in mapping ψ distribution at single-base resolution for low abundant RNA species such as mRNA. In addition, the quantitative feature of BID-seq allows monitoring changes in ψ modification stoichiometry for investigating ψ dynamics in different biological systems.

In this protocol, we detail the application of BID-seq to polyA^+^ RNA purified from mESC, with 3 biologically independent replicates. Different from our previously reported criteria for ψ detection ^9^, we further refine the criteria for ψ detection into a p-value-based version that takes into account the gap ratio, read depth, and the relative relationship between these two parameters in two libraries of both ‘BID-seq’ and ‘Input’. After realignment, the total number of gaps $\left(\sum g_{i}\right)$ and total reads depth $\left(\sum d_{i}\right)$ from all ‘Input’ replicates are compared with the total number of gaps $\left(\sum g_{t}\right)$ and total reads depths $\left(\sum d_{t}\right)$ from all ‘BID-seq’ replicates. The sites with a p-value less than 10^−4^ (*P* < 0.0001, One-sided Fisher’s Exact Test) are selected as ψ candidate sites. Then, three additional filters are employed to minimize the false positives: total reads depth above 20 in both ‘BID-seq’ and ‘Input’ libraries $\left(\sum d_{i}>20\;\text{\&}\sum d_{t}>20\right)$; the average gap count above 5 in ‘BID-seq’ library $\left(\bar{g_{t}}>5\right)$; and the average deletion ratio in ‘BID-seq’ library above 2% $\left(\frac{\sum g_{t}}{\sum d_{t}}>0.02\right)$ and 2-fold higher than that in the ‘Input’ library $\left(\frac{\sum g_{t}}{\sum d_{t}}>\frac{\sum g_{i}}{\sum d_{i}}\right)$.

BID-seq maps mRNA ψ distributions in polyA^+^ RNA purified from mammalian cell lines and tissues, offering stoichiometric information at the modified sites. To further identify the specific ‘writer’ proteins that may install individual ψ sites in mRNA, individual PUS enzyme knockout cell lines could be constructed, followed by a comprehensive mapping of mRNA pseudouridines *via* BID-seq. Besides, starting with other RNA species as the input RNA, like rRNA-depleted total RNA, AlkB-treated small RNAs (size below 200 nt), or chromosome-associated RNA (caRNA), etc., BID-seq can be applied to study ψ profiles in non-coding RNA, repeats RNA, and a series of small RNAs like tRNA, snRNA, snoRNA, etc.

### Comparison with other methods

In CMC-based methods ^4–6^, bioinformatic analysis to rank the quality of RT truncations or false positive truncations caused by RNA decay or RNA fragmentation has been challenging, resulting in a modest number of ψ sites identified in human mRNA with few overlapping sites among different datasets ^28^. An azide-modified CMC has been used to enrich ψ-containing mRNA fragments for NGS sequencing (CeU-seq) ^7^, exhibiting many more detected ψ sites, but it still lacks stoichiometry information at the modified sites. HydraPsi-seq ^8^ was developed to detect and quantify ψ modifications on rRNA and yeast mRNA. Although as low as 10–50 ng input RNA could be used, this method has yet to be broadly applied to map ψ transcriptome-wide in mammalian mRNA. Taken together, a base-resolution and quantitative sequencing approach to accurately map transcriptome-wide ψ distribution has been lacking.

Both RBS-seq ^24^ and BID-seq ^9^ map deletion signatures occurring at ψ sites; however, BID-seq bisulfite selectively converts ψ sites into Ψ-BS adduct without causing any C to U conversions, inducing higher deletion rates than RBS-seq at Ψ sites. Given that deletion signatures are rare in naturally transcribed RNAs, the background signals in BID-seq are much lower than sequencing methods that rely on RT-generated truncation signatures such as ψ-seq and Pseudo-seq. For transcriptome-wide ψ mapping, the comparison of BID-seq and other previously published NGS-based methods is summarized below in Table 1.

Besides NGS-based methods, ψ nanopore sequencing methods have also been developed in the past several years ^29,30^. A direct site-by-site comparison with our results is currently not possible in the absence of ψ nanopore sequencing data for mESC mRNA. The signal-to-noise ratio in the ψ nanopore sequencing is relatively high, creating a hurdle for detecting Ψ sites of low modification fractions. BID-seq can detect ψ in short and structured RNAs. NGS based method is till cheaper comparing with nanopore. Nanopore sequencing method does offer a major advantage in sequencing the full length of RNA and can reveal modifications on one long transcript. We think BID-seq and nanopore sequencing method can be complementary.

### Limitations of BID-seq

In this protocol, sufficient reads coverage is required to detect lowly modified ψ sites on lowly expressed RNAs, with an average of 80 M NGS sequencing depth typically required for each library. When one ψ site is located within three or more uridines, it is difficult to determine the exact ψ-modified site because the same deletion pattern can be generated with ψ at any site. But in these cases, CMC-based RT stop ^4–6^ can be employed to validate the exact ψ site, if necessary. The BID-seq protocol requires ~10 ng polyA^+^ RNA (from ~500 ng cellular total RNA) for library preparation, which needs to be purified from at least ~20,000 cells; however, BID-seq is currently unable to start with input RNA less than 10 ng.

### Experimental design

#### Isolation of polyA^+^ RNA from cultured cells or animal tissues

Both cultured cells and frozen tissue samples can be used for RNA preparation in BID-seq. Tissues should be weighed and homogenized in a proper volume of TRIzol reagent until no visible chunks are left.

After suspending cultured cells or tissues in TRIzol reagent, cellular total RNA can be isolated according to the TRIzol manufacturer’s protocol, followed by isopropanol precipitation. Then, polyA-tailed RNA can be purified by two rounds of polyA^+^ enrichment with the Dynabeads^™^ mRNA Purification Kit (for mRNA purification from total RNA preps).

In BID-seq, please prepare 3 or more biologically independent replicates for each sample. The current BID-seq protocol for library preparation is robust and can be readily reproduced, without any batch-to-batch variations observed. For the controls in library preparation, since a small percentage of rRNA always remains in the purified polyA^+^ RNA, the Ψ profiles at the rRNA can be used as the internal controls for monitoring BID-seq library quality, which typically exhibits very high and consistent deletion ratios among replicates. So, the current protocol does not require additional controls like synthetic spike-in oligos containing ψ.

#### RNA fragmentation and end repair

In BID-seq protocol, up to 48 libraries (24 ‘BID-seq’ and 24 ‘Input’) can efficiently be prepared in one batch, which means we could start with up to 24 polyA^+^ RNA samples per batch.

The purified polyA^+^ RNA was fragmented to ~50–60 nt using RNA Fragmentation Reagent, starting with ~10 ng polyA^+^ RNA as an input. Then, a typical T4 polynucleotide kinase (PNK) treatment can repair both the 3’- and 5’- ends of the RNA fragments, yielding a hydroxyl group at the 3’-end and a monophosphate group at the 5’-end.

#### RNA ligation

The RNA with repaired 3’- and 5’-ends was used for RNA 3’-adaptor ligation. In this step, a high-efficiency ligation was conducted with the ssDNA adaptors containing a 3’-rApp structure, in the presence of T4 RNA Ligase 2 (truncated, KQ). Upon the completion of RNA 3’-adaptor ligation, the excessive 3’-adaptors were removed by the treatments of 5’-deadenylase and RecJf. The complete digestion of the 3’-adaptor is necessary for avoiding adaptor dimers during library construction.

The following RNA 5’-adaptor ligation employed the typical RNA adaptors with -OH groups at both 5’- and 3’- ends. The T4 RNA Ligase 1 (high concentration) promotes the ligation between the 3’ends of RNA adaptors and the repaired 5’-ends of RNA fragments. After these 2 steps of ligations, the ligated RNAs can be stored in a −80 °C freezer for a week.

The 5’- and 3’- dual ligations ensure the complete read-through at the BS-modified ψ site during RT, because only cDNA containing both 5’- and 3’- adaptors can be further amplified by PCR.

#### BID-seq bisulfite treatment and desulphonation

**Fig. 3 |:**
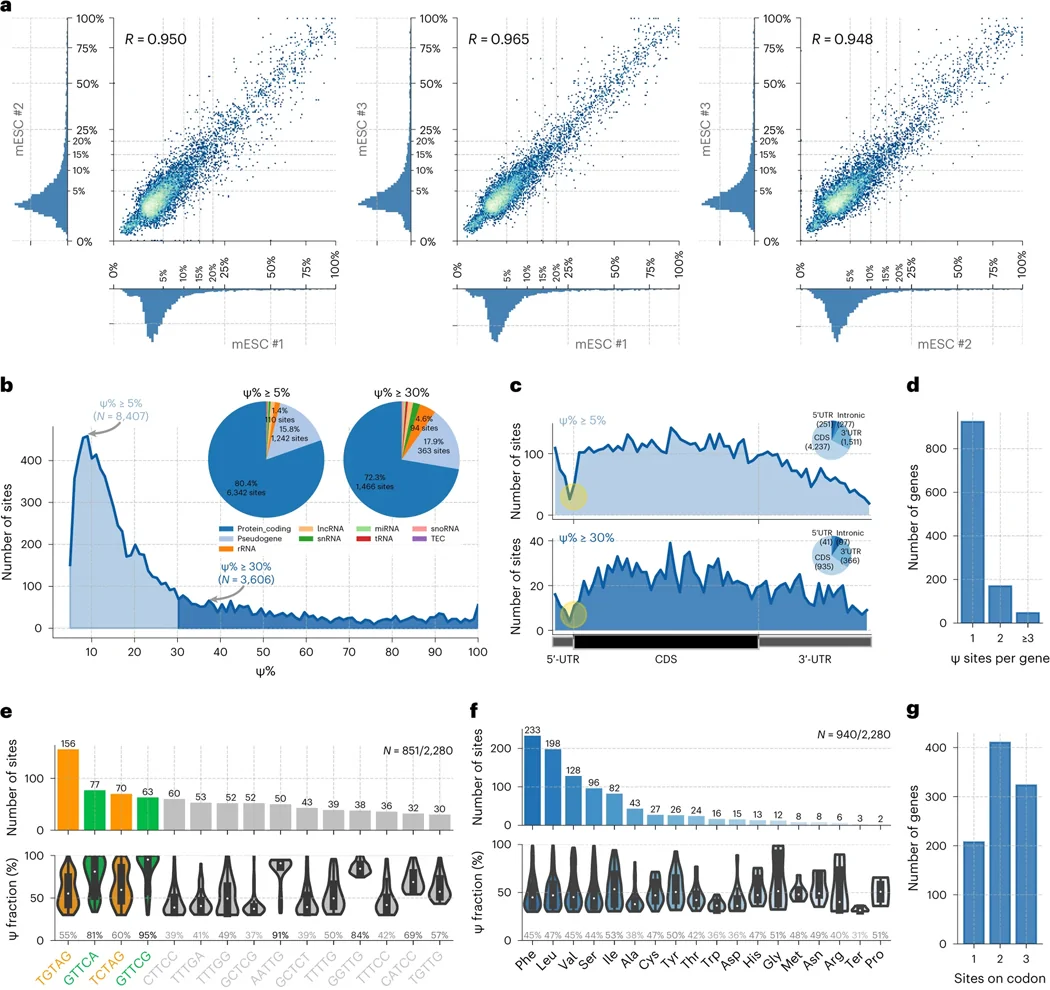
BID-seq reveals thousands of ψ sites in mammalian polyA^+^ RNA. **a**, Among 3 biologically independent replicates of mESC mRNA, BID-seq uncovers thousands of ψ sites with excellent overlap among replicates, through BID-pipe computational pipeline. **b**, BID-seq measures the modification stoichiometry at the identified ψ sites in mESC, with the annotation information of modified RNA species. **c**, The density distribution of ψ sites along three segments of mRNA, including 5’ UTR, CDS, and 3’ UTR. **d**, ψ-modified mRNA mostly contains one single ψ site per gene, in mESC. **e**, The ψ site number and ψ stoichiometry of the top 15 ψ-modified motifs in mESC mRNA, revealed by BID-seq. **f**, The ψ site number and ψ stoichiometry of the frequently modified codons in CDS of mESC mRNA, revealed by BID-seq. **g**, ψ within mRNA CDS mainly occurs at the 2^nd^ nucleotide of the codon, in mESC.

As discussed above in “BID-seq bisulfite chemistry” section, we used 1/6 of ligated RNA as untreated input; meanwhile, we incubated 5/6 of ligated RNA for BID-seq bisulfite treatment at 70 °C for 3 hours. After the bisulfite treatment, we quickly diluted the reaction solution, added RNA binding buffer and ethanol, and mixed well, followed by loading RNAs into the column. Then an on-column desulphonation was conducted and the incubation time has been extended to 75 min in this protocol, which has been confirmed to work robustly in mESC polyA^+^ RNA samples (Fig. 3).

#### Reverse transcription

With the RNAs after bisulfite and desulphonation treatment, we utilized the commercially available SuperScript IV RT to read out Ψ-BS adduct as deletion signatures. We incubated the RT reaction at 50 °C for one hour, as the optimal reaction condition. Right after the RT incubation, a quick RNase H treatment was employed to remove RNA from DNA:RNA hybrid, followed by cDNA purification.

#### NGS sequencing

The polyA^+^ RNAs were fragmented into ~50–60 nt length in “RNA fragmentation and end repair” section. We sequenced all libraries at NovaSeq 6000 in single-end 100 bp mode. In this way, we the sequencer can read through the target region that corresponds to the ~50–60 nt insertion of RNA fragments.

#### Data analysis

The data analysis pipeline for BID-seq is designed for capturing Ψ-induced deletion signatures across the transcriptome, and it has been further improved in this protocol, termed BID-pipe (Fig. 2a). After the adaptor trimming and filtering of low-quality reads, we constructed the pipeline for reads alignment and reads re-alignment. Both ‘BID-seq’ and ‘Input’ samples were processed and analyzed in parallel using BID-pipe, and all features of sequencing reads were comprehensively considered in a p-value-based analysis model. This new pipeline comprehensively takes into account the sequencing depth and deletion rate at each candidate site, allowing for the transcriptome-wide Ψ detection.

## Materials

### Biological materials

- Any mammalian cells or frozen tissue. We used mES cells (ES-E14TG2a, ATCC, cat. no. CRL-1821, https://scicrunch.org/resolver/CVCL_9108). CAUTION: The cell lines used for BID-seq should be regularly checked to ensure they are not infected with mycoplasma.

### Reagents

- UltraPure DEPC-treated water (Thermo Fisher Scientific, cat. no. 750023)
- RNase AWAY^™^ Surface Decontaminant (Thermo Scientific^™^, cat. no. 7005–11)
- TRIzol reagent (Invitrogen, cat. no. 15596026). CAUTION: TRIzol Reagent is hazardous and should be handled in a fume hood.
- Chloroform, pure (CHCl_3_; Fisher Chemical, cat. no. C606–1). CAUTION: Chloroform is hazardous and should be handled in a fume hood.
- Ethyl alcohol, pure (CH_3_CH_2_OH; Sigma-Aldrich, cat. no. E7023)
- 2-Propanol, pure ((CH_3_)_2_CHOH; Fisher Chemical, cat. no. A451–4)
- Dynabeads^™^ mRNA Purification Kit (for mRNA purification from total RNA preps, Invitrogen, cat. no. 61006)
- RNA Clean & Concentrator-5 (Zymo Research, cat. no. R1016)
- DNA Clean & Concentrator-5 (Zymo Research, cat. no. D4014)
- Qubit dsDNA HS Assay Kit (Invitrogen, cat. no. Q32854)
- Qubit RNA HS Assay Kit (Invitrogen, cat. no. Q32852)
- RNA 3′ SR Adaptor (5′App-NNNNN AGATCGGAAGAGCACACGTCT-3SpC3) (Integrated DNA Technologies)
- RNA 5′ SR Adaptor (5′-GUUCAGAGUUCUACAGUCCGACGAUC-3′) (Integrated DNA Technologies)
- SR RT Primer (5′- AGACGTGTGCTCTTCCGATCT −3′) (Integrated DNA Technologies)
- SR PCR Primer (5′-AATGATACGGCGACCACCGAGATCTACACGTTCAGAGTTCTACAGTCCGA- 3′) (Integrated DNA Technologies)
- RNA Fragmentation Reagents (Invitrogen, cat. no. AM8740)
- T4 Polynucleotide Kinase Reaction Buffer (700 mM Tris-HCl, pH 7.6,100 mM MgCl_2_, 50 mM DTT; New England BioLabs, cat. no. B0201S).
- T4 Polynucleotide Kinase (10 U/μL) (Thermo Scientific, cat. no. EK0032)
- SUPERase•In RNase Inhibitor (Invitrogen, cat. no. AM2696)
- T4 RNA Ligase 2, truncated KQ (New England BioLabs, cat. no. M0373L)
- PEG 8000 (50% (wt/vol), included in New England BioLabs, cat. nos. M0373L)
- 5′ Deadenylase (New England BioLabs, cat. no. M0331S; Do not mix this enzyme with RNase-free water directly, to avoid deactivation of the enzyme)
- RecJf exonuclease (New England BioLabs, cat. no. M0264L)
- Deoxynucleotide (dNTP) Solution Mix (New England BioLabs, cat. no. N0447L)
- T4 RNA Ligase 1 (ssRNA Ligase), High Concentration (New England BioLabs, cat. no. M0437M)
- SuperScript^™^ IV Reverse Transcriptase (Invitrogen, cat. no. 18090050)
- RNase H (New England BioLabs, cat. no. M0297L)
- RNaseOUT Recombinant Ribonuclease Inhibitor (Invitrogen, cat. no. 10777019)
- Sodium sulphite (Na_2_SO_3_; Sigma Aldrich, cat. no. 31454–500G)
- Sodium bisulphite (NaHSO_3_; Fisher Chemical, cat. no. S654–500g)
- Desulphonation Buffer (Zymo Research, cat. no. R5001–3-40)
- NEBNext^®^ Multiplex Oligos for Illumina^®^ (Index Primers Set 1) (New England BioLabs, cat. no. E7335S)
- NEBNext^®^ Multiplex Oligos for Illumina^®^ (Index Primers Set 2) (New England BioLabs, cat. no. E7500S)
- NEBNext^®^ Multiplex Oligos for Illumina^®^ (Index Primers Set 3) (New England BioLabs, cat. no. E7710S)
- NEBNext^®^ Multiplex Oligos for Illumina^®^ (Index Primers Set 4) (New England BioLabs, cat. no. E7730S)
- LongAmp^®^ *Taq* 2X Master Mix (New England BioLabs, cat. no. M0287L)
- Agarose (Low-Melting, Nucleic Acid Recovery/Molecular Biology Grade; Thermo Fisher Scientific, cat. no. BP165–25)
- TAE Buffer (Tris-acetate-EDTA) (50X) (Thermo Scientific^™^, cat. no. B49)
- Gel Loading Dye (6X; New England Biolabs, cat. no. B7021S)
- pBR322 DNA-MspI Digest (New England Biolabs, cat. no. N3032S)

### Equipment

- 50 mL round-bottom flask (Synthware)
- 250 mL Erlenmeyer flask (Synthware)
- 100 mL separation funnel (Synthware)
- NanoDrop 8000 Spectrophotometer (Thermo Fisher Scientific, cat. no. ND8000)
- 1.5 mL low-adhesion microcentrifuge tubes (USA Scientific, cat. No. 1415–2600)
- Tubes and domed caps, strips of eight (Thermo Fisher Scientific, cat. no. AB0266)
- Reach Olympus Premium Barrier Tips (Genesee Scientific, cat. no. 10 μL: 23–401; 200 μL: 23–412; 1,000 μL: 23–430)
- ART Barrier Speciality Pipette tips (Thermo Fisher Scientific, cat. no. 2149)
- Pipetman L single-channel pipette (Gilson, P2L, cat. nos. P2L, P10L, P20L, P100L, P200L and P1000L: SKU: FA10001M–10006M)
- Fisherbrand Elite Multichannel Pipettes (Fisher Scientific, cat. no. 1–10 μL: FBE1200010; 30–300 μL: FBE1200030)
- 12-Tube magnetic separation rack (New England BioLabs, cat. no. S1509S)
- Qubit 2.0 Fluorometer (Invitrogen, cat. no. Q33216)
- Qubit assay tubes (Invitrogen cat. no. Q32856)
- 2100 Bioanalyzer Instrument (Agilent, cat. no. G2939BA)
- NovaSeq 6000 System (Illumina)
- BID-seq analysis pipeline (https://github.com/y9c/pseudoU-BIDseq)

### Reagent setup

Reagents flagged as RNase-free should be prepared under RNase-free conditions. Clean gloves and working area with 70% (vol/vol) EtOH and RNase AWAY^™^ Surface Decontaminant. Use RNase-free water when indicated.

#### RNA 3′ SR Adaptor (5′App-NNNNN AGATCGGAAGAGCACACGTCT-3SpC3)

This ssDNA oligonucleotide contains a 5′ adenylation modification (code /5rApp/ in IDT catalog) and a 3′ spacer (code /3SpC3/ in IDT catalog). The probe should be ordered with RNase-free HPLC purification. Dissolve the oligonucleotide in RNase-free water to produce a 20 μM stock solution. Split the stock solution into multiple aliquots and keep it at −80 °C. The dissolved adaptor is stable at −80 °C for at least 1 year.

#### RNA 5′ SR Adaptor (5′-GUUCAGAGUUCUACAGUCCGACGAUC- 3′)

This ssRNA oligonucleotide contains -OH group at both 5’- and 3’-ends. The probe should be ordered with RNase-free HPLC purification. Dissolve the oligonucleotide in RNase-free water to produce a 10 μM stock solution. Split the stock solution into multiple aliquots and keep it at −80 °C. The dissolved adaptor is stable at −80 °C for at least 1 year.

#### SR RT Primer (5′-AGACGTGTGCTCTTCCGATCT- 3′)

This ssDNA oligonucleotide should be ordered with RNase-free HPLC purification. Dissolve the oligonucleotide in RNase-free water to produce a 100 μM stock solution. Dilute and aliquot the stock solution into 50 μL (2 μM) format. Store the aliquots and remaining stock solution at −20 °C. The dissolved primer is stable at −20 °C for at least 1 year.

#### SR PCR Primer (5′-AATGATACGGCGACCACCGAGATCTACACGTTCAGAGTTCTACAGTCCGA - 3′)

This ssDNA oligonucleotide should be ordered with HPLC purification. Dissolve the oligonucleotide in RNase-free water to produce a 100 μM stock solution. Dilute and aliquot the stock solution into 50 μL (10 μM) format. Store the aliquots and remaining stock solution at −20 °C. The dissolved primer is stable at −20 °C for at least 1 year.

## Procedure

### RNA sample preparation

Timing: ~2 hours

1. Prepare 3 or more biologically independent replicates for each sample. For cultured cells and frozen tissue samples (at least 50,000 cells), purify total RNA with a proper amount of TRIzol Reagent and the typical RNA precipitation using chloroform and 2-propanol, according to the manufacturer’s protocol. The current protocol does not require additional controls like synthetic spike-in oligos containing Ψ, since the Ψ profiles at the rRNA can be used as the internal controls for monitoring BID-seq library quality.
2. Enrich mRNA from total RNA with two rounds of polyA-enrichment using Dynabeads mRNA Purification Kit (Invitrogen), according to the manufacturer’s instructions.

**CRITICAL** RNA sample preparation needs to be carried out RNase-free conditions. Clean gloves and working area, with 70% (vol/vol) EtOH and RNase AWAY^™^ Surface Decontaminant. Use RNase-free water and RNase-free EP tubes.

### Library preparation

#### RNA fragmentation

Timing: ~30 mins

- Preheat a PCR block to 70°C. Dilute 10 ng of each RNA sample generated in **Step 2** in 10 μL of RNase-free water, and transfer these samples to a PCR tube. Make a master mix of RNA fragmentation according to the table below and mix the master mix well by pipetting up and down 20 times. Add the 5 μL master mix into each RNA sample. Then mix well by pipetting 20 times.

Prepare a master mix of RNA fragmentation as follow:

**Table:** 

Reagents	Volume (μL)
10X RNA Fragmentation Reagents	1
RNase-free water	4

**CRITICAL** Although 10ng of RNA is given here, the RNA fragmentation conditions in the next step (70°C for 14 min) are compatible with a broad range of RNA input amounts (from 10 ng to 200 ng).

- Heat the RNA samples to 70°C for 14 min in a preheated PCR block.
- Purify the fragmented RNA with Zymo RNA Clean & Concentrator-5 kit according to the manufacturer’s protocol. Elute RNA with 11 μL RNase-free water to get RNA in 10 μL of RNasefree water.

**Pause point**: The eluted RNA (10 μL) can be stored at −80 °C for one week.

### RNA end repair

Timing: ~1.5 hours

- Preheat a PCR block to 37°C. Make a master mix of RNA end repair according to the table below and mix the master mix well by pipetting up and down 20 times.

Add the 20 μL master mix into each RNA sample from **Step 5**. Mix the stock well by pipetting up and down 20 times.

Prepare the master mix of RNA end repair as follow:

**Table:** 

Reagents	Volume (μL)
10X PNK reaction buffer	3
SUPERNase•In^™^	1.5
RNase-free water	12.5
T4 PNK	3

- Heat the samples at 37°C for 45 min in the preheated PCR block.
- Add 1.5 μL T4 PNK and 1.5 μL 10 mM ATP to the samples. Mix the stock well by pipetting 20 times.
- Heat the samples at 37 °C for 45 min in the preheated PCR block.
- Purify samples using the Zymo RNA Clean & Concentrator-5 kit according to the manufacturer’s protocol. Elute RNA with 11 μL RNase-free water to get 3′- and 5′-repaired RNA in 10 μL RNase-free water.

**Pause point**: The eluted RNA (10 μL) can be stored at −80 °C for a week.

### RNA 3′ adaptor ligation

Timing: ~12 hours

- Preheat a PCR block to 70°C. Mix 10 μL of the 3′- and 5′-repaired RNA fragments prepared in **Step 10** with 1 μL of the 20 μM RNA 3′ SR Adapter (5′App-NNNNN AGATCGGAAGAGCACACGTCT-3SpC3). Heat at 70 °C for 2 mins and then immediately place tubes on ice.

**Note**: Placing the samples immediately onto the ice after heating is important, to avoid the denatured RNA gradually renaturing its secondary structures.

- Make a master mix of RNA 3’ ligation according to the table below and mix the master mix well by pipetting up and down 20 times.

Add 11 μL of master mix into each RNA–adapter sample. Mixing each sample again by pipetting up and down 10 times. Then add 2 μL T4 RNA Ligase 2 truncated KQ to each sample, and mix each sample well by pipetting 20 times.

Prepare the master mix of RNA 3’ ligation as follow:

**Table:** 

Reagents	Volume (μL)
10X T4 RNA Ligase Reaction Buffer 500 mM Tris–HCl, pH 7.5, 100 mM MgCl_2_, and 10 mM DTT	2.5
50% PEG8000	7.5
SUPERase•In^™^	1

**CRITICAL** Mixing the master mix of RNA 3’ ligation is important if there are multiple samples. Pipetting up and down 20 times is required for all mixing operations in this step due to the high viscosity of PEG8000. In this step, it is acceptable to add T4 RNA ligase 2 truncated KQ to all samples before starting to mix each individual sample.

- Incubate the samples in a PCR block at 25 °C for 2 h, then 16 °C for 10 h. Put the samples on ice immediately upon the completion of incubation.
- Add 23 μl RNase-free water to each sample and then add 2 μL 5′-deadenylase. Mix the samples well by pipetting 20 times.
- Incubate the samples at 30 °C for 45 min in the preheated PCR block for 5’-deadenylation of excessive adapters.
- Add 1 μL RecJf to each sample for ssDNA digestion. Mix the samples well by pipetting up and down 20 times.
- Incubate the samples at 37 °C for 45 min in the preheated PCR block.
- Purify samples using the Zymo RNA Clean & Concentrator-5 kit according to the manufacturer’s protocol. Elute RNA with 11 μL RNase-free water to get 3′-end-ligated RNA in 10 μL RNase-free water.

**Pause point**: The eluted RNA (10 μL) can be stored at −80 °C for a week.

### RNA 5′ adaptor ligation

Timing: ~8 hours

- Preheat a PCR block to 70°C. Mix the purified 3′-end-ligated RNA from **Step 18** with 1.2 μL 10 μM 5′ SR Adapter (5′-GUUCAGAGUUCUACAGUCCGACGAUC-3′). Heat at 70 °C for 2 mins and immediately place the tubes on ice.

**Note**: Placing the samples immediately onto the ice after heating is important, to avoid the denatured RNA gradually renaturing its secondary structures.

- Make a master mix of RNA 5’ ligation according to the table below and mix the master mix well by pipetting up and down 20 times.

Add 18 μL of master mix into each RNA–adapter sample. Mixing each sample again by pipetting up and down 10 times. Then add 1 μL T4 RNA Ligase 1 to each sample, and mix each sample well by pipetting 20 times.

**? TROUBLESHOOTING**

Prepare the master mix of RNA 5’ ligation as follow:

**Table:** 

Reagents	Volume (μL)
10X T4 RNA Ligase Reaction Buffer	3
50% PEG8000	12
10 mM ATP	3

- Incubate samples at 25 °C for 8 h in a PCR block. Put the samples on ice immediately upon the completion of incubation.
- Purify samples using the Zymo RNA Clean & Concentrator-5 kit according to the manufacturer’s protocol. Elute RNA with 11 μL RNase-free water to get RNA in 10 μL RNase-free water.

**Pause point**: The eluted RNA (10 μl) can be stored at −80 °C for a week.

### BID-seq bisulfite treatment and desulphonation

Timing: ~3 hours for bisulfite treatment + 75 min for desulphonation

- Save a 1.5 μL aliquot of the purified RNA from **Step 22**, labeled as “*Input*” samples. Dilute the “*Input*” sample to 10 μL with RNase-free water and keep on ice.
- Preheat a PCR block to 70°C. Prepared BID-seq BS reagent (2.4 M Na_2_SO_3_ and 0.36 M NaHSO_3_) by dissolving 270 mg Na_2_SO_3_ and 34 mg NaHSO_3_ in 900 μl RNase-free water, and vortex the stock vigorously. Mix the remaining 8.5 μL of RNA from **Step 23** with 45 μL of the freshly prepared BID-seq BS reagent. Mix each sample well by pipetting up and down 20 times.

**? TROUBLESHOOTING**

- Incubate the reaction mixture at 70 °C for 3 h in a preheated PCR block.
- Add 75 μL RNase-free water, 270 μL RNA Binding Buffer (from Zymo RNA Clean and Concentrator kit), and 400 μl ethanol to the reaction mixture sequentially. Mix well by inverting the tubes 15 times and load onto an RNA Clean & Concentrator-5 column.
- Centrifuge the RNA Clean & Concentrator-5 columns at 12,000 x g for 1 min at room temperature. After centrifuge, add 200 μL RNA Wash Buffer (RNA Clean & Concentrator) to each column and centrifuge at 12,000 x g for 1 min at room temperature. Add 200 μL RNA Desulphonation Buffer to the column and then incubate at room temperature for 75 min. Keep the ‘Input’ samples on ice for 75 min when conducting the 75-min desulphonation incubation for the ‘Treated’ samples.
- Add 700 μL RNA Wash Buffer (RNA Clean & Concentrator) to each column and centrifuge at 12,000 x g for 1 min at room temperature. Then, add 700 μL RNA Wash Buffer to each column again and centrifuge at 12,000 x g for 2 min at room temperature. Elute RNA with 11 μL RNase-free water, with a centrifuge at 12,000 x g for 1 min at room temperature. The eluted RNA in 10 μL RNase-free water is labeled as “*Treated*” samples.

**Note**: The “*Treated*” samples cannot be stored in freezers, so the RT should be conducted immediately.

### Reverse transcription and RNase H treatment

Timing: ~1 hour + 30 min

- Preheat a PCR block to 70°C. Add 1.0 μL 2.0 μM SR RT primer (5′-AGACGTGTGCTCTTCCGATCT −3′) to both ‘*Input*’ and ‘*Treated*’ samples. Heat at 65 °C for 2 mins and then immediately place tubes on the ice.

**Note**: Placing the samples immediately onto the ice after heating is important, to avoid the denatured RNA gradually renaturing its secondary structures.

- Preheat a PCR block to 70°C. Prepare a master mix for RT as below:

**Table:** 

Reagents	Volume (μL)
5X SSIV Buffer	4
10 mM dNTP Solution Mix	2
DTT (100 mM)	1
RNaseOUT Recombinant Ribonuclease Inhibitor	0.5
SuperScript IV RT	1 (200 Units)
RNase-free water	0.5

Mix the master mix by pipetting up and down 20 times, then add 9 μL of this master mix to each RNA-primer mixture. Mix well again by pipetting 20 times.

- Incubate the RNA-primer mixture with the master mix prepared in **Step 30** at 50 °C for 1 h in the preheated PCR block.

**Note**: 50°C is the optimal temperature for the highest deletion rates at Ψ-modified sites, whereas 55°C leads to a little bit lower deletion ratio. The 1-hour incubation time ensures that SuperScript IV RT can better read through Ψ-BS adducts within diverse motif contexts.

**Pause point**: The RT product can be stored at 4 °C overnight, though it is not recommended to store it for a longer time.

- After the RT reaction, move the tubes onto ice. Add 1 μL RNase H and mix well by pipetting 20 times.
- Incubate at 37 °C for 20 min, and then heat at 70 °C for 5 min, using the PCR program.
- Purify cDNA samples with Zymo DNA Clean & Concentrator kit according to manufacturer’s instructions. Add a 7X volume (147 μL) of DNA binding buffer and mix by pipetting 15 times. Use 21 μL RNase-free water to elute the cDNA, which should give 20 μL RNase-free water.

**Pause point**: The eluted cDNAs (20 μL) can be stored at −80 °C for up to 6 months.

### PCR amplification for NGS sequencing

Timing: ~4 hours

- For each cDNA sample obtained from **Step 34**, use 4 μL of the cDNA for 15-cycle PCR amplification reaction and save the rest 16 μL at −80 °C for future usage. Prepare the PCR reaction mixture for each sample, according to the table below.

**Note**: To avoid mistakes in assigning indexes for multiple samples, it is recommended to add indexed primers into PCR tubes before the addition of cDNA.

**Table:** 

Reagents	Volume (μL)
cDNA of each individual sample	4
Indexed primer from NEBNext^®^ Multiplex Oligos for Illumina^®^ (One specific index for each individual sample)	1.5
SR PCR Primer	1.5
LongAmp Master Mix	25
RNase-free water	18

- Perform PCR on the samples from **Step 35** according to the settings below:

**Table:** 

Cycle number	Denature	Anneal	Extend
1	94 °C 30s	NA	NA
2–16	94 °C 15s	62 °C 30s	70 °C 30s
17	NA	NA	70 °C 5min

- After PCR amplification, move the sample onto ice. Add 10 μL Gel Loading Dye (6X) to each sample and mix well by pipetting 10 times.
- Make 1X TAE Buffer by diluting the 50X TAE Buffer. Prepare a 3.5% (wt/vol) low melting point agarose gel using 1X TAE Buffer and load 40 μL mixture of each sample (from **Step 37**) into each well. Run at 90 V for 45 mins. Use pBR322 DNA-MspI Digest (NEB) as a size marker.
- Cut the bands at ~175–185 bp using a scalpel, to collect ‘*Treated*’ and ‘*Input*’ samples (example gels are shown in Supplementary Fig. 3a).

**CRITICAL** These library sizes observed by agarose gel indicated the corresponding sizes of RNA fragments produced in **Step 3**; the RNA fragmentation step at 70°C for 14 min gives RNA fragments of a size around 50–60 nt.

**? TROUBLESHOOTING**

**Pause point**: The gel can be stored inside EP tubes at −80 °C overnight; it is not recommended to store it for a longer time.

- Recover DNA from the agarose gel pieces using the MinElute Gel Extraction Kit (QIAGEN), according to the manufacturer’s protocol. Elute the DNA with 20 μL RNase-free water.

**? TROUBLESHOOTING**

- Confirm library sizes using the Bioanalyzer measurement (Supplementary Fig. 3b and 3c). The expected sizes of ‘BID-seq’ and ‘Input’ libraries should be ~175–185 bp.

**? TROUBLESHOOTING**

### Sequencing of BID-seq libraries

- For each sample in **Step 40**, we typically harvest 20 μL eluted DNA at ~1–10 ng/μL, and then submit 10 μL to the sequencing facility. Sequence all libraries on Illumina Nova-seq 6000, with single-end 100 bp read length. Sequence each ‘*Treated*’ sample at ~80–100M reads per library; Meanwhile, sequence each ‘*Input*’ sample at ~30–40M reads per library.

**CRITICAL** The detection limit of the high throughput sequencing method mainly depends on the sequencing depth. For example, for a Ψ site with a modification level of 1%, it becomes challenging to determine whether it is modified or not when the sequencing coverage is only 20 reads around that site; however, with a coverage of 1000 times or more around the site, a more definitive conclusion can be made.

### Sequencing data processing and mutation calling

Timing: ~4 hours

**CRITICAL** The code for sequencing processing and mutation calling has been packaged into one App (docker image), and all the analysis can be achieved by running one line of code. The source code is publicly available on GitHub (https://github.com/y9c/pseudoU-BIDseq).

- Define sample information and customize pipeline settings in one configure file, which is in YAML format. The default configure file is data.yaml under the current directory. Only two sections, ‘reference’ and ‘samples’ are required. Other sections are optional, and if no customized values are provided, the default settings will be used. The important parameters of the data.yaml file are shown in the table below:

**Table:** 

Parameter				Description
Level 1	Level 2	Level 3	Default value (type)	
workdir	-	-	workspace (string)	(*optional*) The path to the working directory.
cores	-	-	48 (int)	(*optional*) Max number of cores for all the running jobs at the same time.
reference	contamination	fa	- (string)	(*optional*) Reference file for putative contamination in the samples
		bt2	Auto (string)	(*optional*) bowtie2 index for contamination reference
	genes	fa	- (string)	(***required***) reference file for selected rRNA, tRNA and snoRNA genes
		bt2	Auto (string)	(*optional*) bowtie2 index for selected genes
	genome	fa	- (string)	(***required***) reference genome for the species you study
		star	- (string)	(***required***) STAR index for reference genome
sample	(sample name)	data	- (list)	(***required***) list of files path for sequencing reads in fastq format. Multiple sequencing run for one sample is supported. You do not need to combine the input fastq files before passing the data into the pipeline.
		group	- (string)	(***required***) group ID for this sample
		treated	true (boolean)	(*optional*) true for BS treated sample, false for untreated control sample.
	(…)	(...)	(…)	(*optional*) other samples. Adding any number of entries is supported.

- Run the whole pipeline with one command (apptainer run docker://y9ch/bidseq). You can specify the version of the pipeline by adding version number into the docker image (eg, docker://y9ch/bidseq:v1). If the configure file is in another name rather than data.yaml, you can specify the configure file with an additional argument (-c filename_of_configure.yaml). Tools used within the pipeline are listed in the table below:

**Table:** 

Tool	Version^*^	Source code	Description
fastp	0.23.2	https://github.com/OpenGene/fastp	Join pair-end sequencing reads
cutadapt	4.1	https://github.com/marcelm/cutadapt	Remove adapter and extract UMI
bgzip (htslib)	1.16	https://github.com/samtools/htslib	Compress txt files
bowtie2 / bowtie2-build	2.5.0	https://github.com/BenLangmead/bowtie2	Map reads against contamination / genes
STAR	2.7.10b	https://github.com/alexdobin/STAR	Map reads against genome
samtools	1.16.1	https://github.com/samtools/samtools	Read, filter and convert mapping results
bedtools	2.30.0	https://github.com/arq5x/bedtools2	Combine position files
UMICollapse	b764b0d	https://github.com/Daniel-Liu-c0deb0t/UMICollapse	Remove duplicates based on UMI
cpup	0.1.0	https://github.com/y9c/cpup	Count depth and deletion for each site

* Version number has been fixed in release v1 of the pipeline, but might be changed in a future release.

- Review the analysis log and library quality report. The number of reads available after trimming, alignment, and duplicate removal is recorded in log files under report_reads directory. You can extract these numbers to have an overall assessment of library quality.

**? TROUBLESHOOTING**

### Pseudouridine sites detection and filtering

Timing: ~1 hour

CRITICAL ψ site detection, filtering and visualization can be achieved within the R language using the criteria described below.

- Detect pseudouridine sites. The TSV files under the pileup_adjusted directory report all sites with a deletion signature in the BID-seq libraries. Within the file, the first 3 columns provide the location of each site, including gene/chromosome ID, position, and strand, respectively. Beginning from the 4^th^ column, sequencing coverage (d) and deletion number (g) are reported for each sample in every two columns. The header line of the file labels the sample names in the same order as the configuration in YAML file. The deletion ratio (r), defined as the deletion number over sequencing coverage (r=gd), allows for the precise calculation of the stoichiometry (f) of the ψ-modified sites. This calculation involves applying the deletion ratio (r) to the calibration curves using the equation:
$$f=\frac{b-r}{c\cdot (b+s-s\cdot r-1)}$$,
  where c is the conversion ratio, s is the RT dropout proportion and b is the background noise. Supplementary Table 1 contains these three parameters for each motif. The dropout proportion in the model corresponds to the RT stop (dropout) ratio of the SuperScript IV RT. Upon bisulfite treatment, the ψ base is converted into a ψ-BS adduct, which is not a natural substrate of SuperScript IV RT and may lead to RT stop. Analysis of the sequencing data from the modified probes showed that the RT enzyme creates more RT stop at several certain sequence contexts. RT stop lowers the sequence coverage and observed deletion ratio. However, by fitting these numbers to the calibration curves, we can well estimate the ψ stoichiometry.
- Filter confident pseudouridine sites by applying cutoffs on sequencing coverage and the deletion ratio which for mESC polyA^+^ RNA are as follows:
  - Total sequencing coverage in ‘BID-seq’ libraries is greater than 20$\left(\sum d_{t}>20\right)$.
  - Total sequencing coverage in ‘Input’ libraries is greater than 20$\left(\sum d_{i}>20\right)$.
  - The average deletion number is above 5 in ‘BID-seq’ library $\left(\bar{g_{t}}>5\right)$.
  - Average deletion ratio in ‘BID-seq’ libraries is above 2%$\left(\frac{\sum g_{t}}{\sum d_{t}}>0.02\right)$.
  - The average deletion ratio in ‘BID-seq’ libraries is 2-fold higher than that in ‘Input’ libraries $\left(\frac{\sum g_{t}}{\sum d_{t}}>\frac{\sum g_{i}}{\sum d_{i}}\right)$.
  - The p-values, from One-sided Fisher’s Exact Test, for the number of T bases in ‘BID-seq’ libraries $\left(\sum d_{t}-\sum g_{t}\right)$, the number of deletions in ‘BID-seq’ libraries $\sum g_{t}$, the number of T bases in ‘Input’ libraries $\left(\sum d_{i}-\sum g_{i}\right)$,and the number of deletions in ‘Input’ libraries ($\sum g_{i}$) are less than 10^−4^.
  - Stoichiometry (f) of the ψ-modified sites is greater than 30% for highly modified sites reported in this protocol (f > 0.3).

**? TROUBLESHOOTING**

## Troubleshooting

Troubleshooting advice can be found in the table below:

**Table:** 

Step	Problem	Problem reason	Solution
20	White precipitate appears in the mixture after adding T4 RNA Ligase 1.	T4 RNA Ligase 1 is used at a high concentration and forms a white precipitate if not mixed immediately when added.	It is important to mix each sample immediately after adding T4 RNA Ligase 1. Mix each sample one by one. Do not add the ligase enzyme to all samples and then start mixing each individual sample. Pipetting up and down 20 times is required for all mixing operations in this step due to the high viscosity of PEG8000.
24	Na_2_SO_3_ and NaHSO_3_ do not dissolve completely.	The high concentration of BID-seq BS reagent (2.4 M Na_2_SO_3_ and 0.36 M NaHSO_3_) leads to a saturated buffer.	Vortex vigorously to dissolve the powder. The BS reagent must be prepared fresh and used immediately in **Step 25**; if the reagent is left at room temperature for more than half an hour some precipitate will appear, and it will no longer be usable. The current BID-seq protocol for library preparation is robust and can be readily reproduced, ideally with the BS reagent always freshly prepared. Vigorous vortexing is necessary to make sure all the chemicals are completely dissolved in water. With the immediate use of the freshly prepared BS reagent, no significant batch-to-batch variations were observed.
39	No notable bands observed on agarose gel.	cDNA was lost during purification after the RT and RNase H treatment in **Step 34**.	In **Step 34**, when using Zymo DNA Clean & Concentrator kit, add a 7X volume of DNA Binding Buffer and mix very well, before loading the mixture to the column. DNA Clean & Concentrator kit can remove short oligos such as RT primers and ligation adapters, to avoid the consumption of PCR primers. The 7X volume of DNA binding buffer can keep all cDNA of ~100 nt. If we add 5X or 2X volume of DNA binding buffer, we will lose most of cDNA and cannot see any bands in **Step 39**.
40	Library concentration >30 ng/μL.	Overamplification.	Keep PCR cycle number no more than 15 cycles.
41	The peak size at BioAnalyzer is much smaller than the expected ~175–185 bp.	Low RNA quality in **Step 3**.	In **Step 3**, if the RNA quality is low or RNA degradation occurred during RNA preparation, you may see smaller peak sizes at BioAnalyzer in **Step 41**. The users should make sure every step is RNase-free when preparing RNA in **Step 3.**
45	Many reads mapped to *E. coli* or mycoplasma	Contamination during cell culture or RNA preparation.	Use sterile reagents and consumables. Check the starting biological samples by RT-qPCR. It is better to have a hood specifically for placing PCR machines.
45	Reads contain high level of duplication.	Overamplification.	Keep PCR cycle number no more than 15 cycles.
45	Many reads mapped to rRNA.	Incomplete removal of rRNA.	Two rounds of polyA-enrichment are needed to remove almost all rRNA, using Dynabeads mRNA Purification Kit. One round of polyA-enrichment is usually not good enough.
47	Notable deletion rate detected at unmodified uridine sites, such as the unmodified uridine sites on rRNA.	Incomplete desulfonation in **Step 27**.	In **Step 27**, after adding 200 μL RNA Desulphonation Buffer to the column, an 75-min incubation is necessary for a complete desulphonation.
47	Low deletion rate detected at RNA ψ sites, such as the benchmark ψ sites on rRNA.	Incomplete BS reaction in **Step 24** and **25**.	In **Step 24**, mix each reaction very well by pipetting 20 times, after adding BID-seq BS reagent to RNA.

## Timing

Steps 1–2, preparation of RNA samples: 2 h

Steps 3–5, RNA fragmentation: 30 min

Steps 6–10, RNA end repair: 1.5 h

Steps 11–18, RNA 3’ adaptor ligation: 12 h

Steps 19–22, RNA 5’ adaptor ligation: 8 h

Steps 23–28, BID-seq bisulfite treatment and desulphonation: 3 h + 75 min

Steps 29–34, Reverse transcription and RNase H treatment: 1 h + 30 min

Steps 35–41, PCR amplification for NGS sequencing: 4 h

Step 42, Illumina sequencing of BID-seq libraries: ~48 hours

Steps 43–45, Sequencing data processing and mutation calling: 4 h

Steps 46–47, Pseudouridine site detection and filtering: 1 h

## Anticipated results

In this protocol, we initially apply an optimized desulphonation treatment and use repeated BID-seq libraries of synthetic RNA oligo probes containing NNUNN (0% modified) and NN ψ NN (100% modified). With the updated BID-seq protocol and BID-pipe analysis, we observe ≤ 1% deletion ratios for 240 out of 256 motifs in the NNUNN probes without any modification, and ≤ 3% deletions at all 256 motifs (Fig. 2c). Meanwhile, for 100% modified NN ψ NN probes, we observe ≥ 50% deletion ratios at 245 out of 256 motifs (Fig. 2d). The background deletions in bisulfite-treated unmodified RNA oligo probes (NNUNN, Fig. 2c) are much lower than those in our previous report ^9^. We next apply the optimized desulphonation treatment and repeated the libraries of calibration curves using synthetic RNA oligo probes containing NNΨNN with varied Ψ fractions. The calibration curves of 256 motif contexts in NN ψ NN are comprehensively shown in Supplementary Fig. 1. We use these probes with varying motif contexts (NN ψ NN) and levels of modification to monitor the deletion ratio (r), and created a model to estimate conversion ratio (c), RT dropout proportion (s) and background noise (b) (Supplementary Table 1). The conversion ratio (c) represents the reactivity of the bisulfite reagent, while the dropout proportion (s) represents the RT stop ratio generated by the SuperScript IV RT. For bisulfite-treated RNA probes containing NN ψ NN with 100% modified ψ, there were 11 sequence contexts exhibiting deletions below 50% (Fig. 2d). Our findings indicate that motifs such as CA ψ AG (Supplementary Fig. 1c), UA ψ UA (Supplementary Fig. 1m), GG ψ UG (Supplementary Fig. 1o), AA ψ CC/CA ψ CC/UA ψ CC (Supplementary Fig. 1f), and AA ψ UU/AG ψ UU/CAΨUU/GA ψ UU/UA ψ UU (Supplementary Fig. 1p) display low deletion ratios at the modified site, as well as high conversion ratios and high dropout proportions. This suggests that the bisulfite reaction did not induce significant biases in these sequence contexts, but the SuperScript IV RT created more RT stop at these motifs, resulting in higher dropout proportions. Considering our current protocol employs dual ligations of a 5’-adaptor and a 3’-adaptor, the cDNAs with an RT stop at the ψ-modified site might not be amplified in the following PCR step, due to the lack of adaptors at both ends. Thus, in these 11 motif contexts, the deletion ratios could be lower because of the increased RT stop. However, with the well-established calibration curves, we can now reasonably estimate ψ modification fractions at these 11 motif contexts.

As ψ sites can only be deposited from U sites by pseudouridine synthase (PUS) enzymes, modifications on A, G, and C bases (such as m^6^A, m^1^A, and m^5^C) will not impact the accuracy of ψ detection. For instance, when examining all m^6^A, m^1^A, and m^5^C sites on mESC rRNA, there were no significant deletion signals observed at these sites (Supplementary Fig. 2a–f). Except for Um modification (2’-*O*-methylation occurring at uridines), other types of RNA modifications on U sites are rare on RNA; for all Um sites on mESC rRNA, the deletion ratios at Um sites were much lower than ψ detection cutoff value used in BID-pipe (Supplementary Fig. 2g-h).

For biological samples, with the current BID-seq protocol, up to 48 libraries (24 ‘BID-seq’ and 24 ‘Input’) can efficiently be prepared within 4 days, excluding the time for RNA purification. With 10 ng of RNA as the input material, the libraries require no more than 15 cycles of PCR amplification to yield a final library concentration of ~10–20 nM. After cutting the target band on agarose gel and purifying the libraries, we obtain a single peak around ~175–185 bp without any detectable adapter dimer peaks < 150 bp (Supplementary Fig. 3), which is ready for NGS sequencing (Fig. 1a).

For the demonstration dataset in this protocol, we performed BID-seq on 3 biologically independent replicates of mESC polyA^+^ RNA, starting from 10 ng RNA. We also prepared 3 corresponding ‘Input’ libraries for each replicate. A total of 108, 97, and 106 million single-end reads were harvested from sequencing the three ‘BID-seq’ libraries, respectively. Then, 54, 46, and 47 million single-end reads were obtained from sequencing the three ‘Input’ libraries. After filtering low-quality reads and adapter dimers, more than 99% of the reads were usable for sequence mapping. 103, 92, and 101 million reads from the ‘BID-seq’ libraries, and 51, 44, and 46 million reads from the ‘Input’ libraries were uniquely mapped to the mouse genome GRCm39. Following de-duplication, 66, 40, and 63 million single-end reads remained for the ‘BID-seq’ libraries, and 37, 25, and 32 million single-end reads remained for the ‘Input’ libraries. Using the new criteria for ψ detection, the extended incubation time of RNA desulphonation, and the new calibration curves built with optimized desulphonation treatment condition, we characterized thousands of ψ sites in polyA^+^ RNA isolated from mESC with much lower background noise, which displayed excellent overlap among 3 biologically independent replicates (the correlation of each pair ranged from 0.948 to 0.965, Fig. 3a). Among 3 replicates of mESC polyA^+^ RNA, we identified 8,407 overlapping ψ sites (above 5% ψ stoichiometry) in mESCs, with 3,606 sites above 30% ψ stoichiometry (Fig. 3b). 6,342 of these 8,407 sites were annotated to mRNA, with 1,466 sites above 30% ψ stoichiometry (Fig. 3b). All these mRNA ψ sites (either >5% or >30% ψ stoichiometry) exhibit a flat distribution across the 5’ UTR, CDS, and 3’ UTR, but the lowest ψ intensity appears near the start codon (Fig. 3c). Although we did not observe such a ψ distribution pattern around start codons in A549 cells previously ^9^, this difference could be attributed to the optimized library preparation protocol, new calibration system, updated BID-pipe analysis pipeline, and inherent differences between A549 cells and mESCs. Like our previous findings in human cell lines ^9^, ψ-modified mRNA from mESCs mostly carry one single Ψ site per gene (Fig. 3d). We then performed analysis on motif context around ψ sites and observed UGΨAG, GUΨCA, UCΨAG, and GUΨCG as frequently modified ones, which are predicted to be substrates of the pseudouridine synthases Pus7 and Trub1 ^9,27^ (Fig. 3e). For ψ deposition within mRNA CDS, tRNA codons of *Phe*, *Leu*, *Val*, *Ser*, and *Ile* are the most frequently ψ-modified codons in mESC mRNA (Fig. 3f), with ψ deposition most likely to occur at the 2^nd^ position of the codon (Fig. 3g).

In this protocol, we replaced the previous cutoff-based method ^9^ with a newly developed p-value-based approach. This new pipeline comprehensively takes into account the sequencing depth and deletion rate at each candidate site, allowing for the identification of transcriptome-wide confident ψ sites. The analysis using the p-value-based approach improves the distinction between lowly modified ψ sites and unmodified uridine sites, which was also assisted by the removal of the background deletions observed in unmodified RNA probes (Fig. 2c). The current BID-seq protocol for library preparation is robust and can be readily reproduced. No significant batch-to-batch variations were observed.

## Supplementary Material

Sup Info

Sup Tables

Sup Report

## Figures and Tables

**Table 1.** Comparison of BID-seq and previous sequencing methods for mapping RNA pseudouridines.

Method	Base-resolution?	Quantitative?	Which base is to be detected?	Detection signature	Chemicals applied	Applicable to mammalian mRNA?	Input RNA amount	Pulldown enrichment?
ψ-seq^4^	Yes	No	ψ	RT stop	CMC-based	Yes	Not specified	No
Pseudo-seq^5^	Yes	No	ψ	RT stop	CMC-based	Yes	polyA^+^ RNA from 2~10 mg total RNA	No
PSI-seq^6^	Yes	No	ψ	RT stop	CMC-based	No	3 mg polyA^+^ RNA	No
CeU-seq^7^	Yes	No	ψ	RT stop	CMC-based	Yes	10 mg polyA^+^ RNA	Yes
RBS-seq^24^	Yes	No	ψ, m^5^C, m^1^A	Deletion	Bisulfite	Yes	5 mg polyA^+^ RNA	No
HydraPsi-seq^8^	Yes	Yes	ψ	Resistant to hydrazine/aniline cleavage	Hydrazine/ aniline	No	10~50 ng polyA^+^ RNA	No
BID-seq^9^	Yes	Yes	ψ	Deletion	Bisulfite	Yes	10 ng polyA^+^ RNA	No

## Data Availability

The underlying data of Fig. 2, Fig. 3, and Supplementary Fig. 1 are deposited into the Gene Expression Omnibus (GEO) database (https://www.ncbi.nlm.nih.gov/geo) under the accession number GSE221976.

## Key Points

- This protocol describes a method for detecting and quantifying pseudouridine residues across the transcriptome.
- The protocol improves on previous pseudouridine sequencing methods by allowing for quantitative base-resolution pseudouridine mapping while covering the entire transcriptome.
