# Supplementary material for: BID-seq for transcriptome-wide quantitative sequencing of mRNA pseudouridine at base resolution
Source: Nat Protoc. Author manuscript; Available in PMC 2025 Feb 1. (PMC11007761; doi:10.1038/s41596-023-00917-5)
Supplement: Supplementary file 1 [file NIHMS1965945-supplement-Sup_Info.pdf]

nature protocols
https://doi.org/10.1038/s41596-023-00917-5

Protocol

Check for updates

BID-seq for transcriptome-wide quantitative sequencing of mRNA pseudouridine at base resolution

In the format provided by the authors and unedited

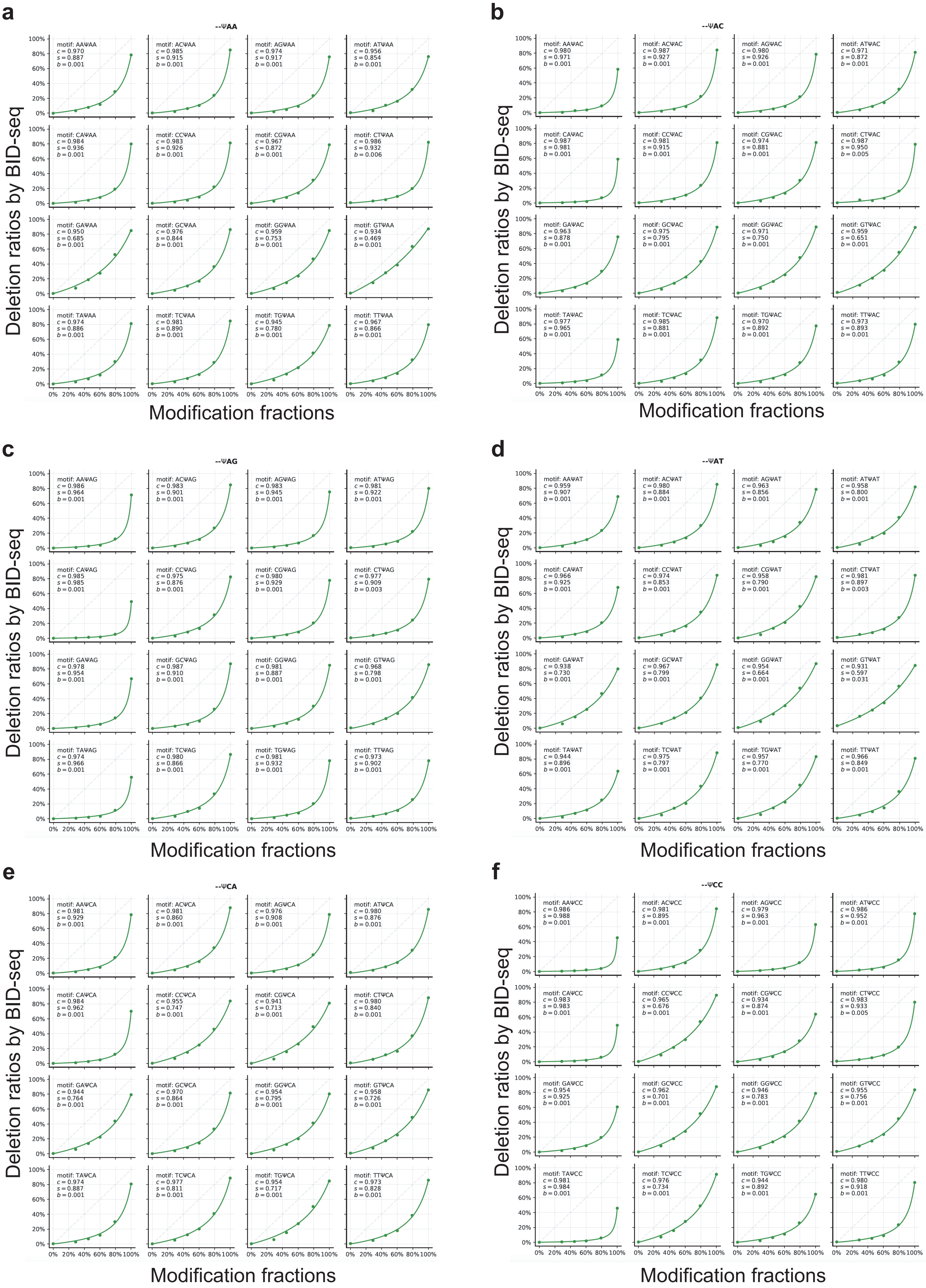
a
--ΨAA
motif: AAΨAA c = 0.970 s = 0.887 b = 0.001
motif: ACΨAA c = 0.985 s = 0.915 b = 0.001
motif: AGΨAA c = 0.974 s = 0.917 b = 0.001
motif: ATΨAA c = 0.956 s = 0.854 b = 0.001
motif: CAΨAA c = 0.984 s = 0.936 b = 0.001
motif: CCΨAA c = 0.983 s = 0.926 b = 0.001
motif: CGΨAA c = 0.967 s = 0.872 b = 0.001
motif: CTΨAA c = 0.986 s = 0.932 b = 0.006
motif: GAΨAA c = 0.950 s = 0.685 b = 0.001
motif: GCΨAA c = 0.976 s = 0.844 b = 0.001
motif: GGΨAA c = 0.959 s = 0.753 b = 0.001
motif: GTΨAA c = 0.934 s = 0.469 b = 0.001
motif: TAΨAA c = 0.974 s = 0.886 b = 0.001
motif: TCΨAA c = 0.981 s = 0.890 b = 0.001
motif: TGΨAA c = 0.945 s = 0.780 b = 0.001
motif: TTΨAA c = 0.967 s = 0.866 b = 0.001
Deletion ratios by BID-seq
Modification fractions
0% 20% 40% 60% 80% 100%
b
--ΨAC
motif: AAΨAC c = 0.980 s = 0.971 b = 0.001
motif: ACΨAC c = 0.987 s = 0.927 b = 0.001
motif: AGΨAC c = 0.980 s = 0.926 b = 0.001
motif: ATΨAC c = 0.971 s = 0.872 b = 0.001
motif: CAΨAC c = 0.987 s = 0.981 b = 0.001
motif: CCΨAC c = 0.981 s = 0.915 b = 0.001
motif: CGΨAC c = 0.974 s = 0.881 b = 0.001
motif: CTΨAC c = 0.987 s = 0.950 b = 0.005
motif: GAΨAC c = 0.963 s = 0.878 b = 0.001
motif: GCΨAC c = 0.980 s = 0.795 b = 0.001
motif: GGΨAC c = 0.971 s = 0.750 b = 0.001
motif: GTΨAC c = 0.959 s = 0.651 b = 0.001
motif: TAΨAC c = 0.977 s = 0.965 b = 0.001
motif: TCΨAC c = 0.985 s = 0.881 b = 0.001
motif: TGΨAC c = 0.970 s = 0.892 b = 0.001
motif: TTΨAC c = 0.973 s = 0.893 b = 0.001
Deletion ratios by BID-seq
Modification fractions
c
--ΨAG
motif: AAΨAG c = 0.986 s = 0.964 b = 0.001
motif: ACΨAG c = 0.983 s = 0.901 b = 0.001
motif: AGΨAG c = 0.983 s = 0.945 b = 0.001
motif: ATΨAG c = 0.981 s = 0.922 b = 0.001
motif: CAΨAG c = 0.985 s = 0.985 b = 0.001
motif: CCΨAG c = 0.975 s = 0.876 b = 0.001
motif: CGΨAG c = 0.980 s = 0.929 b = 0.001
motif: CTΨAG c = 0.977 s = 0.909 b = 0.003
motif: GAΨAG c = 0.978 s = 0.954 b = 0.001
motif: GCΨAG c = 0.987 s = 0.910 b = 0.001
motif: GGΨAG c = 0.981 s = 0.887 b = 0.001
motif: GTΨAG c = 0.968 s = 0.798 b = 0.001
motif: TAΨAG c = 0.974 s = 0.966 b = 0.001
motif: TCΨAG c = 0.980 s = 0.866 b = 0.001
motif: TGΨAG c = 0.981 s = 0.932 b = 0.001
motif: TTΨAG c = 0.973 s = 0.902 b = 0.001
Deletion ratios by BID-seq
Modification fractions
d
--ΨAT
motif: AAΨAT c = 0.959 s = 0.907 b = 0.001
motif: ACΨAT c = 0.980 s = 0.884 b = 0.001
motif: AGΨAT c = 0.963 s = 0.856 b = 0.001
motif: ATΨAT c = 0.958 s = 0.800 b = 0.001
motif: CAΨAT c = 0.966 s = 0.925 b = 0.001
motif: CCΨAT c = 0.974 s = 0.853 b = 0.001
motif: CGΨAT c = 0.958 s = 0.790 b = 0.001
motif: CTΨAT c = 0.981 s = 0.897 b = 0.003
motif: GAΨAT c = 0.938 s = 0.730 b = 0.001
motif: GCΨAT c = 0.967 s = 0.799 b = 0.001
motif: GGΨAT c = 0.954 s = 0.664 b = 0.001
motif: GTΨAT c = 0.931 s = 0.597 b = 0.031
motif: TAΨAT c = 0.944 s = 0.896 b = 0.001
motif: TCΨAT c = 0.975 s = 0.797 b = 0.001
motif: TGΨAT c = 0.957 s = 0.770 b = 0.001
motif: TTΨAT c = 0.966 s = 0.849 b = 0.001
Deletion ratios by BID-seq
Modification fractions
e
--ΨCA
motif: AAΨCA c = 0.981 s = 0.929 b = 0.001
motif: ACΨCA c = 0.981 s = 0.860 b = 0.001
motif: AGΨCA c = 0.976 s = 0.908 b = 0.001
motif: ATΨCA c = 0.980 s = 0.876 b = 0.001
motif: CAΨCA c = 0.984 s = 0.962 b = 0.001
motif: CCΨCA c = 0.955 s = 0.747 b = 0.001
motif: CGΨCA c = 0.941 s = 0.713 b = 0.001
motif: CTΨCA c = 0.980 s = 0.840 b = 0.001
motif: GAΨCA c = 0.944 s = 0.764 b = 0.001
motif: GCΨCA c = 0.970 s = 0.864 b = 0.001
motif: GGΨCA c = 0.954 s = 0.795 b = 0.001
motif: GTΨCA c = 0.958 s = 0.726 b = 0.001
motif: TAΨCA c = 0.974 s = 0.887 b = 0.001
motif: TCΨCA c = 0.977 s = 0.811 b = 0.001
motif: TGΨCA c = 0.954 s = 0.717 b = 0.001
motif: TTΨCA c = 0.973 s = 0.828 b = 0.001
Deletion ratios by BID-seq
Modification fractions
f
--ΨCC
motif: AAΨCC c = 0.986 s = 0.988 b = 0.001
motif: ACΨCC c = 0.981 s = 0.895 b = 0.001
motif: AGΨCC c = 0.979 s = 0.963 b = 0.001
motif: ATΨCC c = 0.986 s = 0.952 b = 0.001
motif: CAΨCC c = 0.983 s = 0.983 b = 0.001
motif: CCΨCC c = 0.965 s = 0.676 b = 0.001
motif: CGΨCC c = 0.934 s = 0.874 b = 0.001
motif: CTΨCC c = 0.983 s = 0.933 b = 0.005
motif: GAΨCC c = 0.954 s = 0.925 b = 0.001
motif: GCΨCC c = 0.962 s = 0.701 b = 0.001
motif: GGΨCC c = 0.946 s = 0.783 b = 0.001
motif: GTΨCC c = 0.955 s = 0.756 b = 0.001
motif: TAΨCC c = 0.981 s = 0.984 b = 0.001
motif: TCΨCC c = 0.965 s = 0.734 b = 0.001
motif: TGΨCC c = 0.944 s = 0.892 b = 0.001
motif: TTΨCC c = 0.980 s = 0.918 b = 0.001
Deletion ratios by BID-seq
Modification fractions

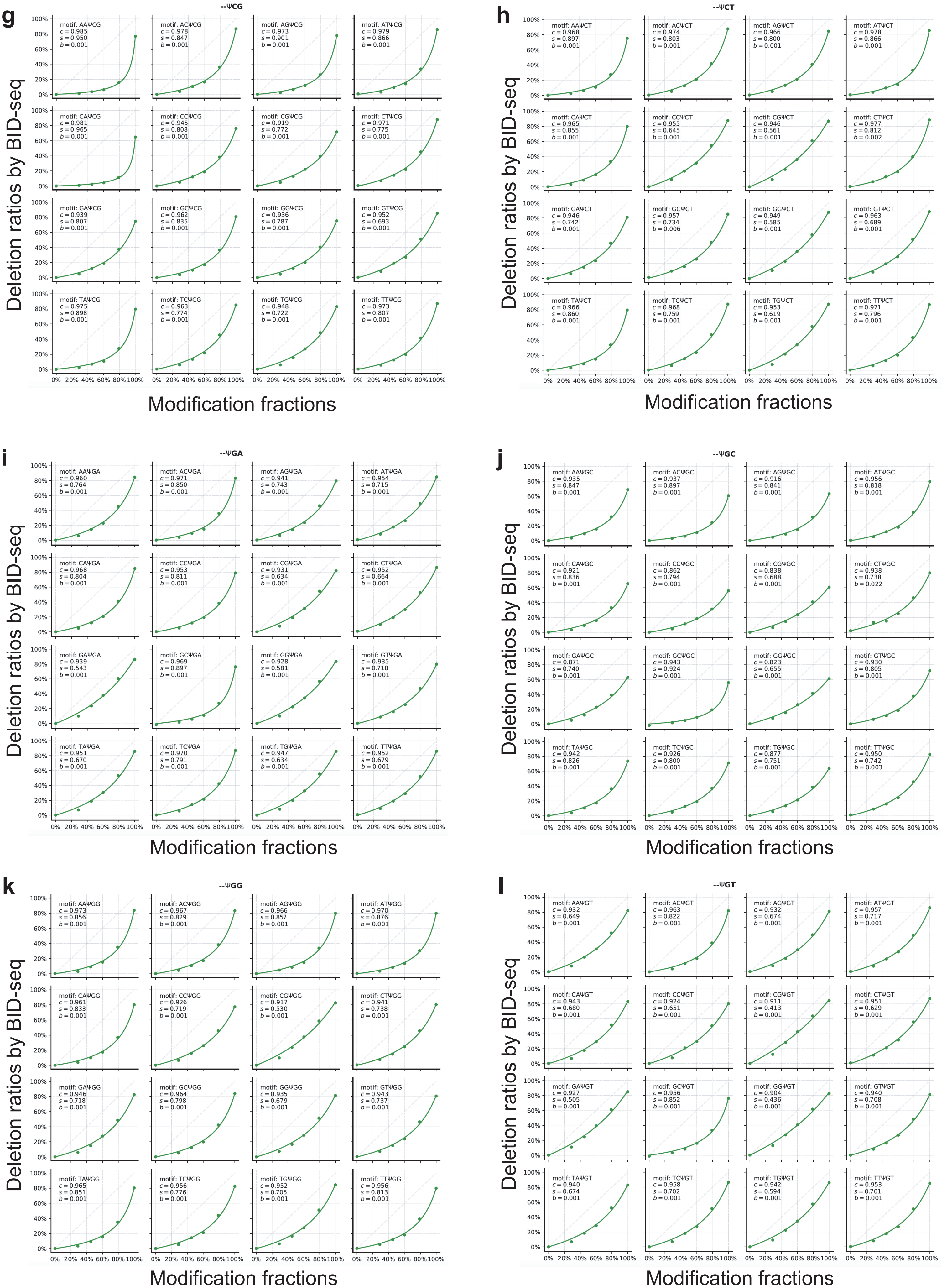
g
--ΨCG
motif: AAΨCG c = 0.985 s = 0.950 b = 0.001
motif: ACΨCG c = 0.978 s = 0.847 b = 0.001
motif: AGΨCG c = 0.973 s = 0.901 b = 0.001
motif: ATΨCG c = 0.979 s = 0.866 b = 0.001
motif: CAΨCG c = 0.981 s = 0.965 b = 0.001
motif: CCΨCG c = 0.945 s = 0.808 b = 0.001
motif: CGΨCG c = 0.919 s = 0.772 b = 0.001
motif: CTΨCG c = 0.971 s = 0.775 b = 0.001
motif: GAΨCG c = 0.939 s = 0.807 b = 0.001
motif: GCΨCG c = 0.962 s = 0.835 b = 0.001
motif: GGΨCG c = 0.936 s = 0.787 b = 0.001
motif: GTΨCG c = 0.952 s = 0.693 b = 0.001
motif: TAΨCG c = 0.975 s = 0.898 b = 0.001
motif: TCΨCG c = 0.963 s = 0.774 b = 0.001
motif: TGΨCG c = 0.948 s = 0.722 b = 0.001
motif: TTΨCG c = 0.973 s = 0.807 b = 0.001
Deletion ratios by BID-seq
Modification fractions
0% 20% 40% 60% 80% 100%
h
--ΨCT
motif: AAΨCT c = 0.968 s = 0.897 b = 0.001
motif: ACΨCT c = 0.974 s = 0.803 b = 0.001
motif: AGΨCT c = 0.966 s = 0.800 b = 0.001
motif: ATΨCT c = 0.978 s = 0.866 b = 0.001
motif: CAΨCT c = 0.965 s = 0.855 b = 0.001
motif: CCΨCT c = 0.955 s = 0.645 b = 0.001
motif: CGΨCT c = 0.946 s = 0.561 b = 0.001
motif: CTΨCT c = 0.977 s = 0.812 b = 0.002
motif: GAΨCT c = 0.946 s = 0.742 b = 0.001
motif: GCΨCT c = 0.957 s = 0.734 b = 0.006
motif: GGΨCT c = 0.949 s = 0.585 b = 0.001
motif: GTΨCT c = 0.963 s = 0.689 b = 0.001
motif: TAΨCT c = 0.966 s = 0.860 b = 0.001
motif: TCΨCT c = 0.968 s = 0.759 b = 0.001
motif: TGΨCT c = 0.953 s = 0.619 b = 0.001
motif: TTΨCT c = 0.971 s = 0.796 b = 0.001
Deletion ratios by BID-seq
Modification fractions
i
--ΨGA
motif: AAΨGA c = 0.960 s = 0.764 b = 0.001
motif: ACΨGA c = 0.971 s = 0.850 b = 0.001
motif: AGΨGA c = 0.941 s = 0.743 b = 0.001
motif: ATΨGA c = 0.954 s = 0.715 b = 0.001
motif: CAΨGA c = 0.968 s = 0.804 b = 0.001
motif: CCΨGA c = 0.953 s = 0.811 b = 0.001
motif: CGΨGA c = 0.931 s = 0.634 b = 0.001
motif: CTΨGA c = 0.952 s = 0.664 b = 0.001
motif: GAΨGA c = 0.939 s = 0.543 b = 0.001
motif: GCΨGA c = 0.969 s = 0.897 b = 0.001
motif: GGΨGA c = 0.928 s = 0.581 b = 0.001
motif: GTΨGA c = 0.935 s = 0.718 b = 0.001
motif: TAΨGA c = 0.951 s = 0.670 b = 0.001
motif: TCΨGA c = 0.970 s = 0.791 b = 0.001
motif: TGΨGA c = 0.947 s = 0.634 b = 0.001
motif: TTΨGA c = 0.952 s = 0.679 b = 0.001
Deletion ratios by BID-seq
Modification fractions
j
--ΨGC
motif: AAΨGC c = 0.935 s = 0.847 b = 0.001
motif: ACΨGC c = 0.937 s = 0.897 b = 0.001
motif: AGΨGC c = 0.916 s = 0.841 b = 0.001
motif: ATΨGC c = 0.956 s = 0.818 b = 0.001
motif: CAΨGC c = 0.921 s = 0.836 b = 0.001
motif: CCΨGC c = 0.862 s = 0.794 b = 0.001
motif: CGΨGC c = 0.838 s = 0.688 b = 0.001
motif: CTΨGC c = 0.938 s = 0.738 b = 0.022
motif: GAΨGC c = 0.871 s = 0.740 b = 0.001
motif: GCΨGC c = 0.943 s = 0.924 b = 0.001
motif: GGΨGC c = 0.823 s = 0.655 b = 0.001
motif: GTΨGC c = 0.930 s = 0.805 b = 0.001
motif: TAΨGC c = 0.942 s = 0.826 b = 0.001
motif: TCΨGC c = 0.926 s = 0.800 b = 0.001
motif: TGΨGC c = 0.877 s = 0.751 b = 0.001
motif: TTΨGC c = 0.950 s = 0.742 b = 0.003
Deletion ratios by BID-seq
Modification fractions
k
--ΨGG
motif: AAΨGG c = 0.973 s = 0.856 b = 0.001
motif: ACΨGG c = 0.967 s = 0.829 b = 0.001
motif: AGΨGG c = 0.966 s = 0.857 b = 0.001
motif: ATΨGG c = 0.970 s = 0.876 b = 0.001
motif: CAΨGG c = 0.961 s = 0.833 b = 0.001
motif: CCΨGG c = 0.926 s = 0.719 b = 0.001
motif: CGΨGG c = 0.917 s = 0.530 b = 0.001
motif: CTΨGG c = 0.941 s = 0.738 b = 0.001
motif: GAΨGG c = 0.946 s = 0.718 b = 0.001
motif: GCΨGG c = 0.964 s = 0.798 b = 0.001
motif: GGΨGG c = 0.935 s = 0.679 b = 0.001
motif: GTΨGG c = 0.943 s = 0.737 b = 0.001
motif: TAΨGG c = 0.965 s = 0.851 b = 0.001
motif: TCΨGG c = 0.956 s = 0.776 b = 0.001
motif: TGΨGG c = 0.952 s = 0.705 b = 0.001
motif: TTΨGG c = 0.956 s = 0.813 b = 0.001
Deletion ratios by BID-seq
Modification fractions
l
--ΨGT
motif: AAΨGT c = 0.932 s = 0.649 b = 0.001
motif: ACΨGT c = 0.963 s = 0.822 b = 0.001
motif: AGΨGT c = 0.932 s = 0.674 b = 0.001
motif: ATΨGT c = 0.957 s = 0.717 b = 0.001
motif: CAΨGT c = 0.943 s = 0.680 b = 0.001
motif: CCΨGT c = 0.924 s = 0.651 b = 0.001
motif: CGΨGT c = 0.911 s = 0.413 b = 0.001
motif: CTΨGT c = 0.951 s = 0.629 b = 0.001
motif: GAΨGT c = 0.927 s = 0.505 b = 0.001
motif: GCΨGT c = 0.956 s = 0.852 b = 0.001
motif: GGΨGT c = 0.904 s = 0.436 b = 0.001
motif: GTΨGT c = 0.940 s = 0.708 b = 0.001
motif: TAΨGT c = 0.940 s = 0.674 b = 0.001
motif: TCΨGT c = 0.958 s = 0.702 b = 0.001
motif: TGΨGT c = 0.942 s = 0.594 b = 0.001
motif: TTΨGT c = 0.953 s = 0.701 b = 0.001
Deletion ratios by BID-seq
Modification fractions

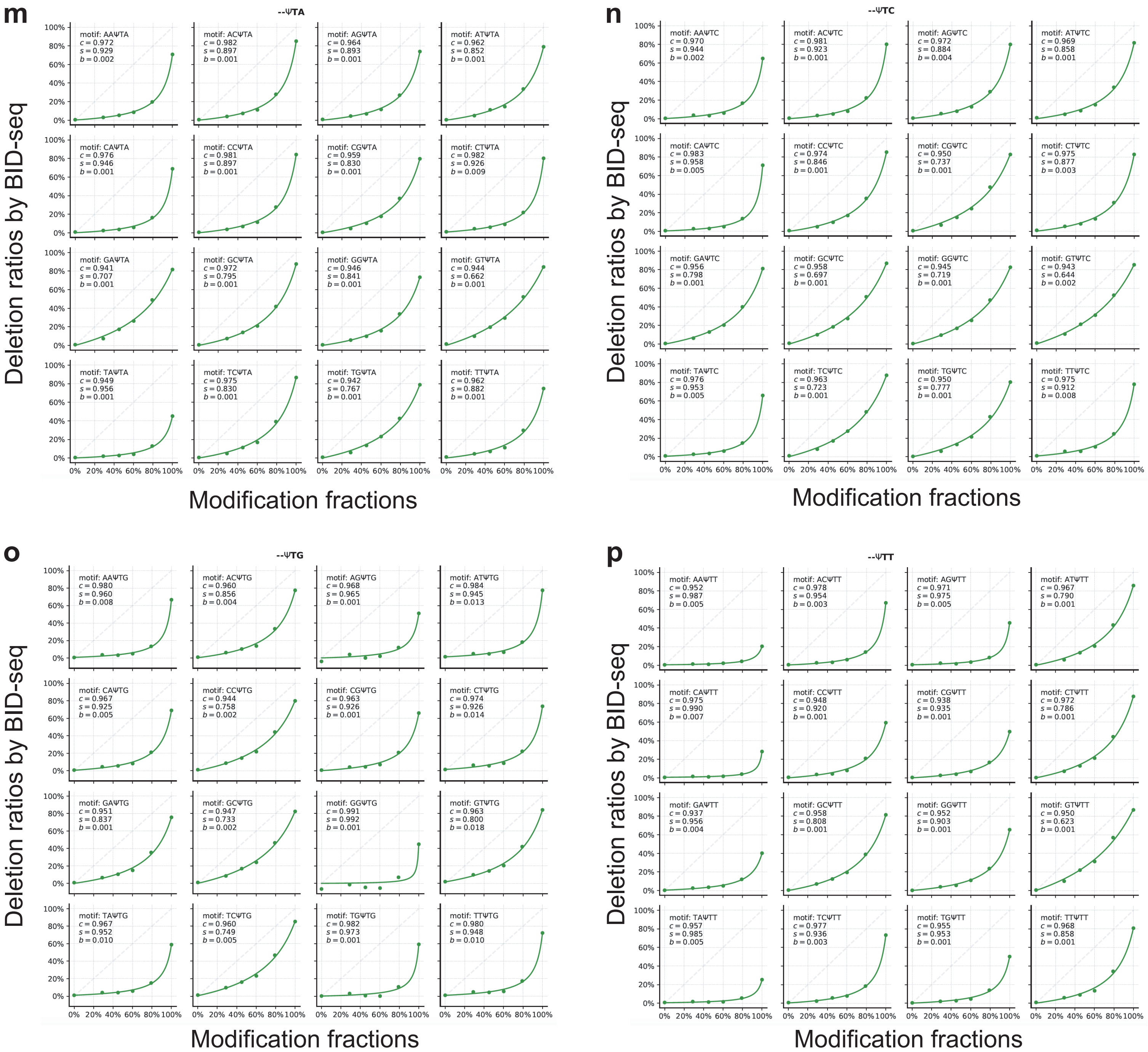

Supplementary Fig. 1 | The calibration curves for BID-seq, based on BID-pipe analysis. a, The BID-seq calibration curves for motif contexts containing NN-ΨAA. **b**, The BID-seq calibration curves for motif contexts containing NN-ΨAC. **c**, The BID-seq calibration curves for motif contexts containing NN-ΨAG. **d**, The BID-seq calibration curves for motif contexts containing NN-ΨAU. **e**, The BID-seq calibration curves for motif contexts containing NN-ΨCA. **f**, The BID-seq calibration curves for motif contexts containing NN-ΨCC. **g**, The BID-seq calibration curves for motif contexts containing NN-ΨCG. **h**, The BID-seq calibration curves for motif contexts containing NN-ΨCU. **i**, The BID-seq calibration curves for motif contexts containing NN-ΨGA. **j**, The BID-seq calibration curves for motif contexts containing NN-ΨGC. **k**, The BID-seq calibration curves for motif contexts containing NN-ΨGG. **l**, The BID-seq calibration curves for motif contexts containing NN-ΨGU. **m**, The BID-seq calibration curves for motif contexts containing NN-ΨUA. **n**, The BID-seq calibration curves for motif contexts containing NN-ΨUC. **o**, The BID-seq calibration curves for motif contexts containing NN-ΨUG. **p**, The BID-seq calibration curves for motif contexts containing NN-ΨUU.

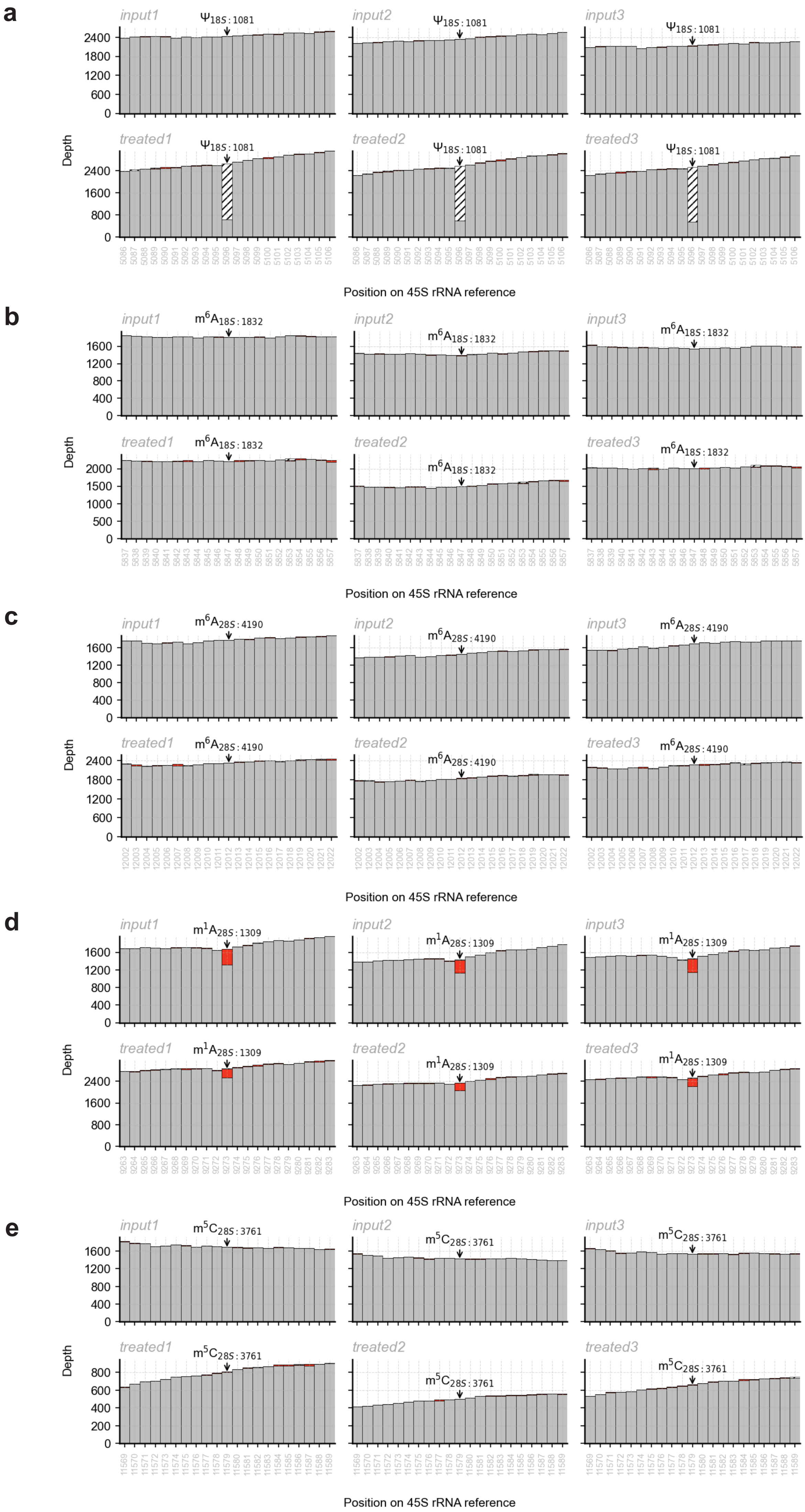

a
input1
input2
input3
treated1
treated2
treated3
Ψ18S:1081
Depth
Position on 45S rRNA reference
b
m6A18S:1832
Depth
Position on 45S rRNA reference
c
m6A28S:4190
Depth
Position on 45S rRNA reference
d
m1A28S:1309
Depth
Position on 45S rRNA reference
e
m5C28S:3761
Depth
Position on 45S rRNA reference

f

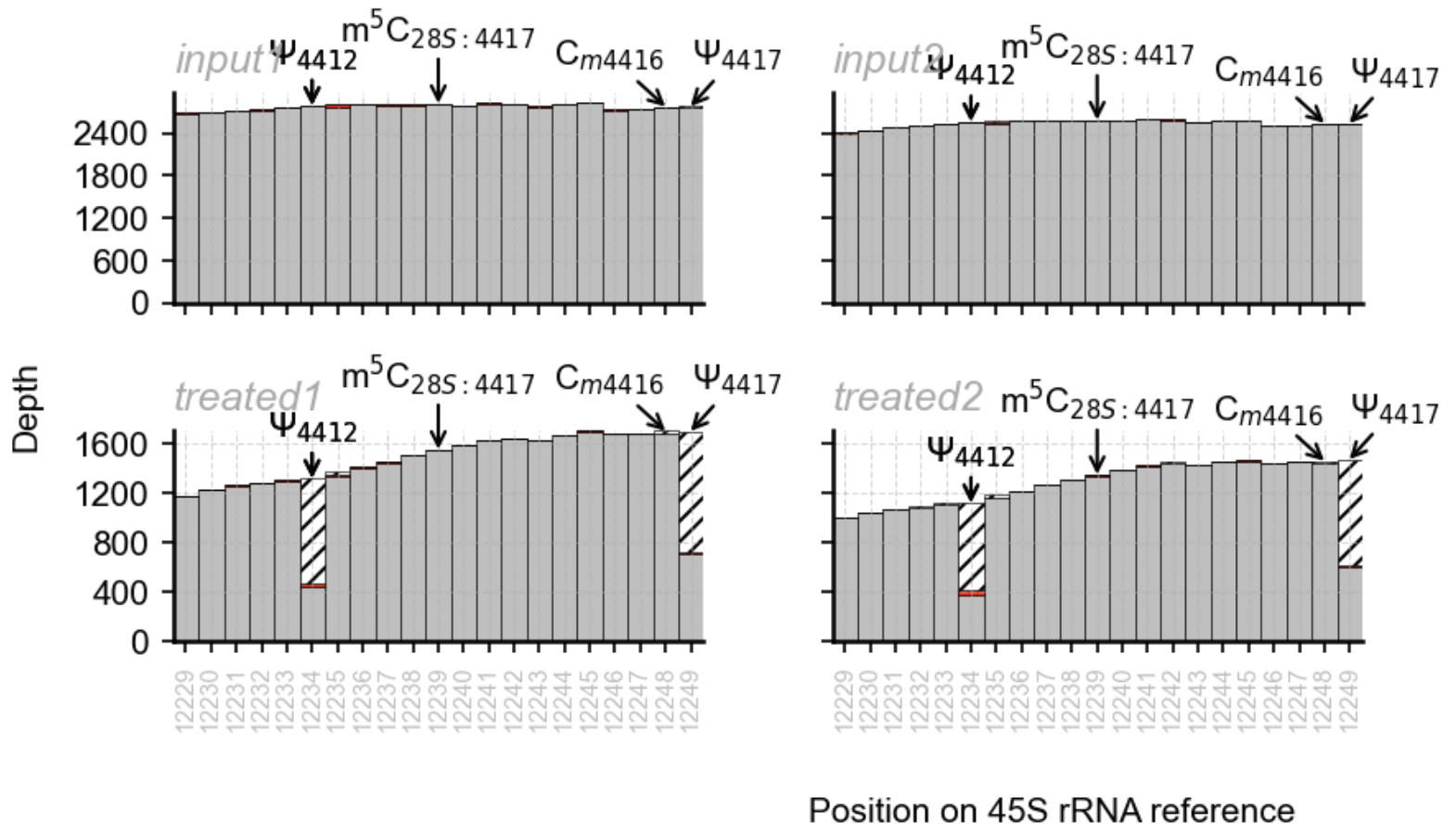

g

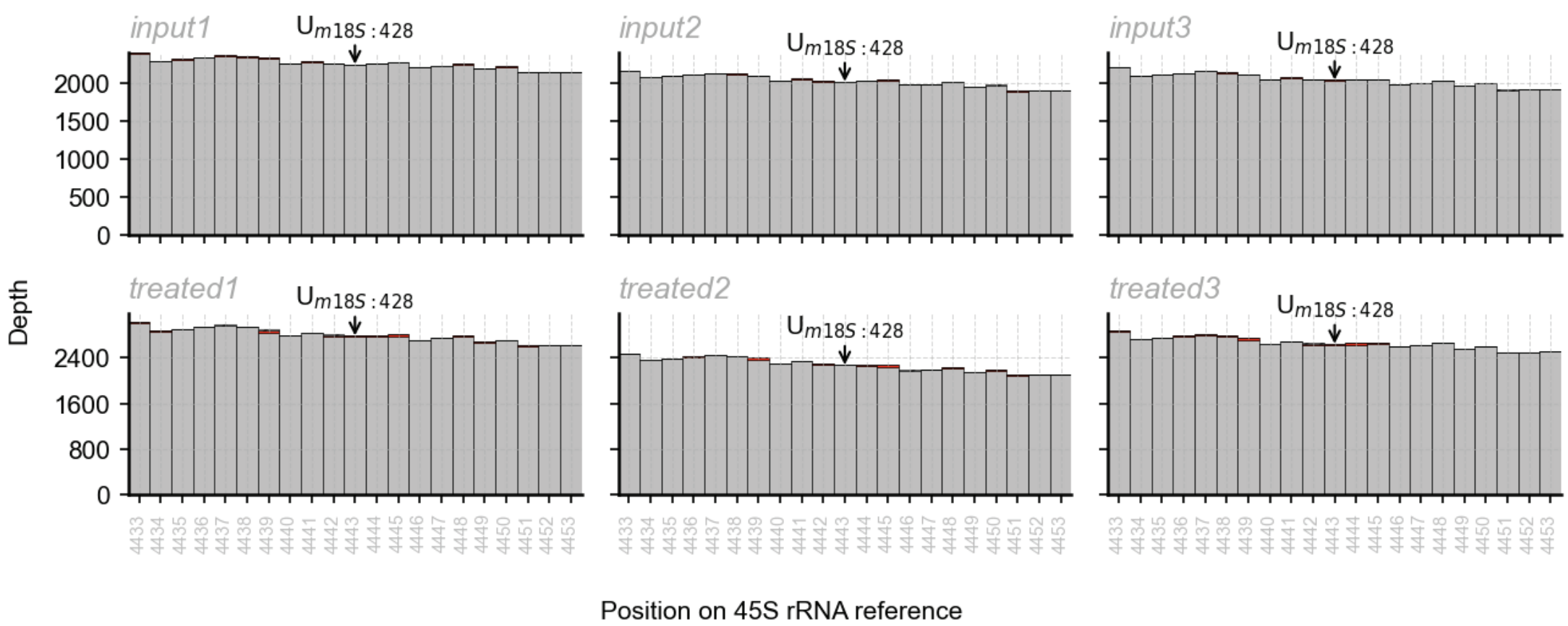

h

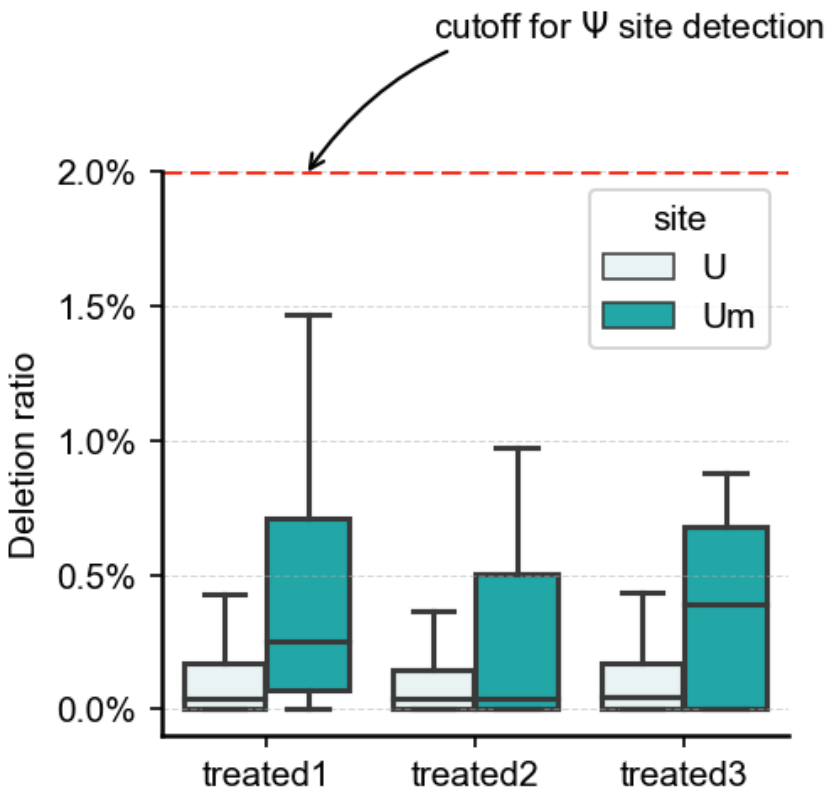

Supplementary Fig. 2 | The deletion profiles of SuperScript IV RT at different RNA modifications on mESC rRNA. **a**, BID-seq reveals dramatic deletion signatures at Ψ1081 of mESC 18S rRNA. **b**, BID-seq does not reveal any deletion signatures at m^6A1832 of mESC 18S rRNA. **c**, BID-seq does not reveal any deletion signatures at m^6A4190 of mESC 28S rRNA. **d**, BID-seq does not reveal any deletion signatures at m^1A1309 of mESC 28S rRNA. **e**, BID-seq does not reveal any deletion signatures at m^5C3761 of mESC 28S rRNA. **f**, BID-seq does not reveal any deletion signatures at m^5C4417 of mESC 28S rRNA. **g**, BID-seq does not reveal any deletion signatures at Um428 of mESC 18S rRNA. **h**, For all Um sites on mESC rRNA, the deletion ratios at Um sites are much lower than Ψ detection cutoff in BID-pipe.

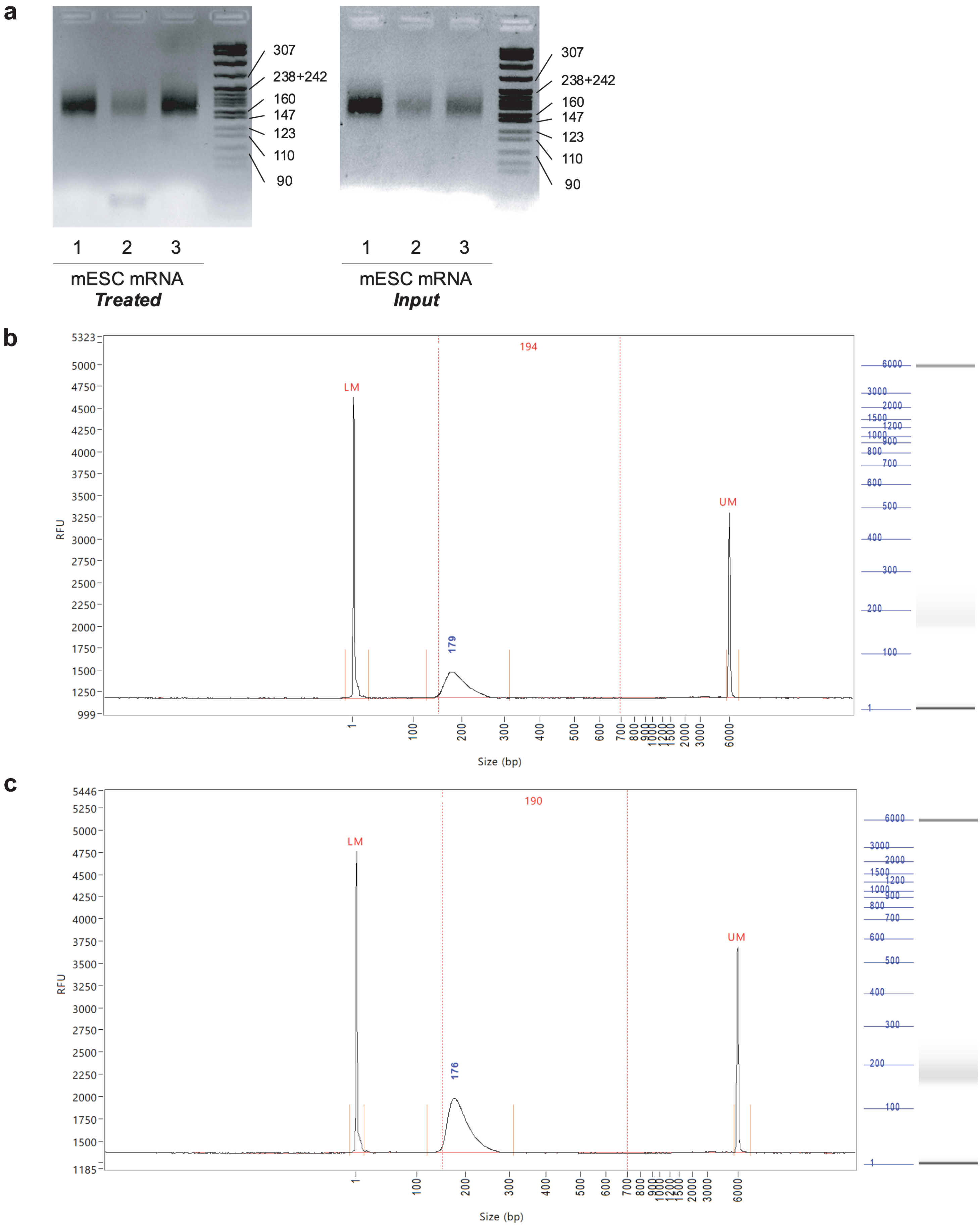

Supplementary Fig. 3 | The Agarose gel and Bioanalyzer plot to show the size of BID-seq libraries. **a**, The agarose gel image to show the sizes of 'Input' and 'Treated' libraries built with mESC polyA$^+$ RNA, in BID-seq. **b**, The Bioanalyzer measurement to show the size of 'Input' library built with mESC polyA$^+$ RNA, in BID-seq. **c**, The Bioanalyzer measurement to show the size of 'Treated' library built with mESC polyA$^+$ RNA, in BID-seq.
